# Chemotaxonomy and Antibacterial Activity of the Extracts and Chemical Constituents of *Psychotria succulenta* Hiern. (Rubiaceae)

**DOI:** 10.1155/2022/7856305

**Published:** 2022-06-15

**Authors:** Darille Claudia Ngnokam Jouogo, Jean-De-Dieu Tamokou, Rémy Bertrand Teponno, Germaine Matsuete-Takongmo, Laurence Voutquenne-Nazabadioko, Léon Azefack Tapondjou, David Ngnokam

**Affiliations:** ^1^Research Unit of Applied and Environmental Chemistry, Department of Chemistry, Faculty of Science, University of Dschang, P.O. Box 67, Dschang, Cameroon; ^2^Research Unit of Microbiology and Antimicrobial Substances, Department of Biochemistry, Faculty of Science, University of Dschang, P.O. Box 67, Dschang, Cameroon; ^3^Groupe Isolement et Structure, Institut de Chimie Moléculaire de Reims (ICMR), CNRS UMR 7312, Bat. 18, B.P. 1039, 51687 Reims Cedex 2, France

## Abstract

The use of natural products for medicinal purposes is becoming more and more common nowadays, as evidenced by the presence in plants of secondary metabolites with different potentials such as antioxidant and antibacterial properties. We evaluated in this work the antimicrobial activities of the extracts and some isolated compounds from the seeds of *Psychotria succulenta* Hiern. (Rubiaceae), a Cameroonian medicinal plant traditionally used to cure microbial infections. The ethanol extract was prepared by maceration and extracted with ethyl acetate and *n*-butanol. The EtOAc (*m* = 168 g) and *n*-BuOH (*m* = 20 g) extracts were further fractionated by silica gel column chromatography to isolation of compounds. Their structures were elucidated by spectroscopic analysis and by comparison with published data. The antibacterial activity of extracts and compounds was assessed by evaluating the minimum inhibitory concentration (MIC) and minimum bactericidal concentration (MBC) against pathogenic bacteria. Thirteen compounds including four alkaloids (veprisine (**1**), naucleofficine III (**2**), vepridimerine B (**3**), and vepridimerine C (**4**)), three triterpenes (barbinervic acid (**5**), 3-*O*-*α*-L-rhamnopyranosyl quinovic acid (**6**), and oleanolic acid (**7**)), one steroid (*β*-sitosterol-3-*O*-*β*-D-glucopyranoside (**8**)), four phenolic compounds (scopoletin (**9**), gallic acid (**10**), quercetin-3-*O*-*β*-D-glucopyranoside (**11**), and kaempferol 3-*O*-*α*-L-rhamnopyranoside-7-*O*-*α*-L-rhamnopyranoside (**12**)), and one iridoid (borreriagenin (**13**)) were isolated from the EtOAc and *n*-BuOH extracts. These compounds were identified by 1D and 2D NMR combined analysis as well as by melting point comparison. The EtOH, EtOAc, and *n*-BuOH extracts exhibited significant antibacterial activities (MIC = 32‐128 *μ*g/mL; MBC = 64‐256 *μ*g/mL) against *Staphylococcus aureus* (Gram-positive bacterium), *Pseudomonas aeruginosa*, *Escherichia coli*, and *Klebsiella pneumonia* (Gram-negative bacteria). Among the isolated compounds, scopoletin (**9**) showed a moderate activity against *Klebsiella pneumoniae* with MIC and MBC values of 16 *μ*g/mL and 32 *μ*g/mL, respectively. It appears that, chemotaxonomically, some of the isolated compounds have already been obtained from the genus *Psychotria* but to the best of our knowledge, this is the first report on the phytochemical investigation of *P. succulenta*. Although many other studies need to be achieved, our results support the use of *P. succulenta* in traditional medicine to cure infectious diseases particularly those caused by the tested bacteria.

## 1. Introduction

Infectious diseases caused by bacteria, viruses, fungi, and other parasites continue to cause enormous damage in the world. Gram-negative or Gram-positive bacteria are able to acquire resistance mechanisms to face environmental aggression (natural environment, competing bacteria, host defense, or antibiotics) either by the modification of sites of action of anti-infective molecules or by production of degradative enzymes. It is therefore important to develop new drugs using natural plants to fight antibiotic resistance and to limit undesirable side effects. *Psychotria succulenta* Hiern. (Rubiaceae) is a shrub of varying size between 1 and 2 m with yellow fruits of ripeness commonly found in most tropical regions [1]. Plants of the genus *Psychotria* (leaves, roots, barks, and rhizomes) are commonly used in traditional medicine for treating bronchial and gastrointestinal disorders [2]. They are also used for curing infections of female reproductive system [3].

To the best of our knowledge, no phytochemical nor pharmacological works have been achieved on *P. succulenta*. Previous works carried out on *Psychotria* species have shown that the different extracts (petroleum ether, chloroform, ethyl acetate, dichloromethane, ethanol, and methanol extracts), the fractions, and some isolated compounds exhibited interesting biological activities such as antibacterial, cytotoxic, antioxidant, antimycobacterial, and antimutagenic properties [4–7]. Plants of this genus are characterized as an abundant source of indole, monoterpene indole, quinoline, and isoquinoline alkaloids as well as flavonoids [8]. In the course of our search of bioactive compounds from some medicinal plants growing in Cameroon [9, 10], we undertook the phytochemical study of *P. succulenta*, leading to the isolation and structure elucidation of thirteen compounds. Furthermore, the crude EtOH extract, the EtOAc and *n*-BuOH extracts as well as some of the isolated secondary metabolites were evaluated for their antibacterial activity, and the results are also presented.

## 2. Materials and Methods

### 2.1. Plant Material

The seeds of *P. succulenta* were collected in October 2018 in Foreke-Dschang (5° 26′ 0^″^ N, 10° 4′ 0^″^ E), West Region of Cameroon, and identified at the National Herbarium of Cameroon by comparison to the voucher specimen deposited under the reference no. 42155/HNC.

### 2.2. Extraction Procedure

The seeds of *P. succulenta* were dried at room temperature and then crushed in fine powder to give 2.8 kg. Two kilograms (2 kg) of this powder was extracted with ethanol (3 × 12 L) for 72 hours to yield 287.2 g of crude ethanol extract after evaporation of the solvent under reduced pressure. A part of this crude extract (250 g) was suspended in distilled water (600 mL), then extracted with EtOAc and *n*-BuOH, respectively. After evaporation of each solvent under reduced pressure, 168 g and 20 g of EtOAc and *n*-BuOH extracts were obtained, respectively.

### 2.3. Isolation of Secondary Metabolites

One hundred and sixty grams (160 g) of the EtOAc extract was subjected to silica gel column chromatography eluted with *n*-hexane-EtOAc (95 : 5 → 20 : 80), then with EtOAc-MeOH (95 : 5 → 80 : 20) to yield five major fractions A-E. Fraction B (18 g) was purified by column chromatography on silica gel eluted with *n*-hexane-EtOAc (80-20) to yield compounds **1** (9 mg), **5** (25 mg), **6** (27 mg), and **7** (20 mg). Fraction C (26 g) was subjected to a silica gel column chromatography eluted with *n*-hexane-EtOAc (30-70) followed by Sephadex LH-20 (Eluted with MeOH) to afford compound **9** (35 mg). Treatment of fraction D (40 g) with column chromatography on silica gel by using *n*-hexane-EtOAc (50-50) as eluent gave compounds **2** (18 mg), **3** (28 mg), and **4** (23 mg). Fraction E (8 g) was chromatographed on Sephadex LH-20 gel column using MeOH as eluent followed by column chromatography on silica gel eluted by EtOAc-MeOH (95-5) to give compounds **8** (33 mg), **10** (22 mg), and **11** (11 mg). An amount of the *n*-BuOH fraction (17 g) was first chromatographed on Sephadex column eluted with MeOH to yield two subfractions coded F and G. The subfraction G was submitted to a silica gel column eluted with EtOAc-MeOH-H_2_O (9-1-1) to give compounds **12** (21 mg) and **13** (15 mg).

### 2.4. General Experimental Procedures

#### 2.4.1. Chromatographic Methods

Column chromatography was carried out on Merck silica gel 60 (70–230 mesh) and gel permeation on Sephadex LH-20, while TLC was carried out on precoated silica gel 60 F_254_ (Merck) plates developed with different solvents and mixture of hexane-EtOAc, EtOAc-MeOH, MeOH-H_2_O, and EtOAc-MeOH-H_2_O. Detection was done by using UV light (254 and 365 nm) and by spraying with 10% H_2_SO_4_ followed by heating at 100°C.

#### 2.4.2. NMR Analysis

The ^1^H and ^13^C-NMR spectra were recorded on a Bruker Avance III 500 spectrometer equipped with a cryoplatform (^1^H at 500 MHz and ^13^C at 125 MHz). 2D NMR experiments were achieved using standard Bruker microprograms (Xwin-NMR version 2.1 software). All chemical shifts (*δ*) are given in parts per million (ppm) with the solvent signal as reference relative to TMS as internal standard, while the coupling constants (*J*) are given in hertz (Hz). Deuterated solvents such as methanol (methanol-*d_4_*), dimethyl sulfoxide (DMSO-*d6*), and chloroform (CDCl_3_) were used as solvents.

### 2.5. Antimicrobial Assay

#### 2.5.1. Microorganisms

The extracts and some isolated compounds were tested for their antibacterial activities against four bacterial strains, namely, *Staphylococcus aureus* ATCC 25923 (Gram-positive bacterium), *Pseudomonas aeruginosa* ATCC 76110, *Escherichia coli* ATCC 25922, and *Klebsiella pneumonia* 22 (Gram-negative bacteria). These microorganisms were taken from the Research Unit of Microbiology and Antimicrobial Substances. The different bacterial species were maintained at +4°C and activated on BBL® nutrient agar (NA, Conda, Madrid, Spain) for 24 h before any antibacterial testing.

#### 2.5.2. Determination of the Inhibition Parameters

The determination of the minimum inhibitory concentration (MIC) was performed using the broth microdilution method [11]. Bacterial suspensions were prepared from the 18-hour-old cultures. Three colonies of the bacterium were then taken and diluted separately with sterile 0.9% NaCl solution to give a turbidity comparable to that of the 0.5 point on the McFarland scale corresponding to approximately 1.5 × 10^8^ CFU/mL. This suspension was again diluted to 1/100 and adjusted to obtain an absorbance of 0.100 at 600 nm corresponding to a bacterial concentration of 10^6^ CFU/mL. Microtiter plates (96 microwells) were made, and each well received 85 *μ*L of Mueller Hinton broth and 5 *μ*L of inoculum. 10 *μ*L of test sample stock solution at a corresponding concentration was then added to each well to reach final concentrations ranging from 0.25 to 256 *μ*g/mL. The positive control was made with the appropriate liquid medium and bacterial suspension only while the negative control was made with 10% DMSO aqueous solution in place of the inoculum. Ciprofloxacin was used as reference antibiotic. The plates were covered and incubated under agitation at 35°C for 24 h. Bacterial growth was determined by introducing 5 *μ*L of a 0.2 mg/mL para-iodonitrotetrazolium solution. Any change in colour from yellow to violet indicates bacterial growth. The minimum inhibitory concentration was defined as the smallest concentration of the substance that prevents this colour change. 10 *μ*L of the contents of each well were aseptically collected and spread separately on the surface of Mueller Hinton agar medium for the purpose of determining the minimum bactericidal concentrations (MBC), which are defined as the smallest concentrations that result in a negative subculture or only one colony. Three replicates were performed for each test sample.

## 3. Results

### 3.1. Chemical Analysis

**Figure 1:**
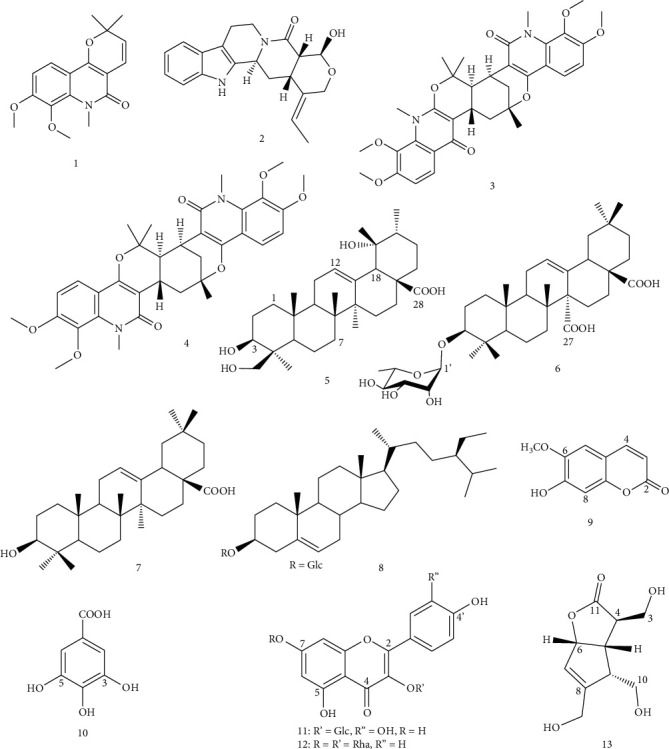
Structures of compounds **1**-**13** isolated from *P. succulenta*. **1**: veprisine; **2**: naucleofficine III; **3**: vepridimerine B; **4**: vepridimerine C; **5**: barbinervic acid; **6**: quinovic acid 3*β*-*O*-*α*-L-rhamnoside; **7**: oleanolic acid; **8**: *β*-sitosterol 3-*O*-*β*-D-glucopyranoside; **9**: scopoletin; **10**: gallic acid; **11**: quercetin 3-*O*-*β*-D-glucopyranoside; **12**: kaempferol 3-*O*-*α*-L-rhamnopyranoside-7-*O*-*α*-L-rhamnopyranoside; **13**: borreriagenin.

The EtOAc and *n*-BuOH extracts from the EtOH crude extract of *P. succulenta* were subjected to repeated column chromatography on silica gel and Sephadex LH-20 to yield thirteen metabolites (**1-13**). Their structures were elucidated by spectroscopic and spectrometric analysis as well as by comparison with literature data (Supplementary materials/figures (available here)). The isolated compounds were identified as veprisine (**1**) [12], naucleofficine III (**2**) [13], vepridimerine B (**3**) [14], vepridimerine C (**4**) [14], barbinervic acid (**5**) [15], quinovic acid 3*β*-*O*-*α*-L-rhamnoside (**6**) [16], scopoletin (**9**) [17], gallic acid (**10**) [18], quercetin 3-*O*-*β*-D-glucopyranoside (**11**) [19], kaempferol 3-*O*-*α*-L-rhamnopyranoside-7-*O*-*α*-L-rhamnopyranoside (**12**) [20], and borreriagenin (**13**) [21]. Compounds **7** and **8** were identified by co-TLC with authentic samples and melting point measurement as oleanolic acid [22] and *β*-sitosterol 3-*O*-*β*-D-glucopyranoside [23], respectively (Figure 1).

***Veprisin (1)***: yellow amorphous powder; ^1^H-NMR (500 MHz, methanol-*d_4_*): *δ* (ppm) = 7.75 (1H, *d*, *J* = 9.0 Hz, H-5), 7.09 (1H, *d*, *J* = 9.0 Hz, H-6), 6.61 (1H, *d*, *J* = 9.9 Hz, H-4′), 5.61 (1H, *d*, *J* = 9.9 Hz, H-3′), 3.98 (3H, s, OCH_3_), 3.91 (3H, s, NCH_3_), 3.80 (3H, s, OCH_3_), and 1.51 (6H, s, CH_3_). ^13^C-NMR (125 MHz, methanol-*d_4_*): *δ* (ppm) = 162.8 (C-1), 156.1(C-7), 155.8 (C-3), 137.0 (C-8), 133.8 (C-9), 125.6 (C-3′), 118.8 (C-5), 116.7 (C-4′), 111.5 (C-4), 108.1 (C-6), 102.9 (C-2), 78.6 (C-3′), 60.4 (OCH_3_), 54.6 (OCH_3_), 33.2 (NCH_3_), and 26.1 (CH_3_)

***Naucleofficine III (2)***: white amorphous powder; ^1^H-NMR (500 MHz, CDCl_3_) *δ* (ppm) = 7.53 (1H, *d*, *J* = 7.7 Hz, H-9), 7.37 (1H, *d*, *J* = 7.9 Hz, H-12), 7.23 (1H, *dd*, *J* = 14.3, 6.8 Hz, H-11), 7.17 (1H, *dd*, *J* = 13.2, 6.3 Hz, H-10), 5.54 (1H, *dd*, *J* = 14.9, 4.8 Hz, H-19), 5.28 (1H, *d*, *J* = 7.5 Hz, H-17), 5.17 (1H, *d*, *J* = 7.7 Hz, H-5a), 5.04 (1H, *m*, H-3), 4.32 (1H, *d*, *J* = 13.1 Hz, H-21a), 4.20 (1H, *d*, *J* = 13.4 Hz, H-21b), 3.04 (1H, *m*, H-4a), 3.02 (1H, *m*, H-5b), 2.77 (1H, *m*, H-4b), 2.74 (1H, *m*, H-15), 2.58 (1H, *m*, H-14a), 2.56 (1H, *t*, *J* = 6.5 Hz; H-16), 2.10 (1H, *m*, H-14b), and 1.62 (1H, *d*, *J* = 11.2 Hz, H-18). ^13^C-NMR (125 MHz, CD Cl_3_): *δ* (ppm) = 169.1 (C-22), 136.0 (C-13), 133.4 (C-20), 133.0 (C-2), 127.4 (C-8), 122.4 (C-11), 122.2 (C-19), 120.2 (C-10), 118.4 (C-9), 111.1 (C-12), 111.0 (C-7), 93.8 (C-17), 68.0 (C-21), 53.0 (C-3), 48.6 (C-16), 42.0 (C-5), 29.2 (C-15), 28.0 (C-14), 21.0 (C-6), and 12.7 (C-18)

***Vepridimerine C (3)***: white amorphous powder; ^1^H-NMR (500 MHz, CDCl_3_): *δ* (ppm) = 8.08 (1H, *d*, *J* = 9.0 Hz, H-4), 7.71 (1H, *d*, *J* = 9.0 Hz, H-13), 6.96 (1H, *d*, *J* = 9.0 Hz, H-3), 6.84 (1H, *d*, *J* = 9.0 Hz, H-12), 3.95 (3H, *s*, 2-OCH_3_), 3.90 (3H, *s*, 10-OCH_3_), 3.77 (3H, *s*, 1*-*OCH_3_), 3.74 (3H, *s*, 11*-*OCH_3_), 3.92 (3H, *s*, NCH_3_), 3.76 (3H, *s*, NCH_3_), 3.96 (1H, *m*, H-19a), 3.20 (1H, q, *J* = 3.2 Hz, H-7), 2.74 (1H, *td*, *J* = 12.7, 4.2 Hz, H-16a), 2.15 (1H, *m*, H-16x), 1.91 (3H, *s*, (CH_3_-6)), 1.72 (3H, *s*, (CH_3_-15)), 1.60 (1H, *dd*, *J* = 12.5, 3.4 Hz, H-6a), 1.48 (1H, *m*, H-16y), 1.34 (1H, *m*, H-19b), and 1.41 (3H, *s*, (CH_3_-6)). ^13^C-NMR (125 MHz, CDCl_3_): *δ* (ppm) = 176.5 (C-17), 163.4 (C-8), 157.7(C-4b), 155.4 (C-2), 155.0 (C-13b), 154.9 (C-10), 137.6 (C-11), 136.7 (C-1), 134.8 (C-18a), 134.1 (C-9a), 122.0 (C-4), 118.8 (C-13), 121.4 (C-4a), 112.7 (C-13a), 112.1 (C-7a), 108.4 (C-3), 107.0 (C-12), 100.1 (C-16b), 84.7 (C-6), 78.5 (C-15), 61.7 (OCH_3_), 61.3 (OCH_3_), 56.3 (OCH_3_), 56.2 (OCH_3_), 52.3 (C-6a), 39.3 (C-19), 35.8 (NCH_3_), 33.8 (NCH_3_), 31.1 (C-16), 29.2 (15-CH_3_), 28.6 (6-CH_3_), 25.7 (C-16a), 25.6 (C-7), and 21.0 (6-CH_3_)

***Vepridimerine B (4)***: white amorphous powder; ^1^H-NMR (500 MHz, CDCl_3_): *δ* (ppm) = 7.70 (1H, *d*, *J* = 9.0 Hz, H-13), 7.66 (1H, *d*, *J* = 9.0 Hz, H-4), 6.88 (1H, *d*, *J* = 9.0 Hz, H-12), 6.84 (1H, *d*, *J* = 9.0 Hz, H-3), 3.98 (3H, *s,* 1-OCH_3_), 3.95 (3H, *s,* 10-OCH_3_), 3.84 (3H, *s,* 2-OCH_3_), 3.76 (3H, *s,* 11-OCH_3_), 3.93 (3H, *s,* NCH_3_), 3.87 (3H, *s,* NCH_3_), 3.84 (2H, *m*, H-19a), 3.24 (1H, q, *J* = 3.2 Hz, H-7), 2.62 (1H, *td*, *J* = 12.7, 4.2 Hz, H-16a), 2.17 (1H, *m*, H-16x), 1.92 (3H, *s*, 6-CH_3_), 1.71 (3H, *s*, 15-CH_3_), 1.59 (1H, *dd*, *J* = 12.5, 3.4 Hz, H-6a), 1.47 (1H, *m*, H-16y), 1.32 (1H, *m*, H-19b), and 1.39 (3H, *s*, (CH_3_-6)). ^13^C-NMR (125 MHz, CDCl_3_): *δ* (ppm) = 164.3 (C-17), 163.3 (C-8), 155.8 (C-4b), 155.0 (C-1), 154.9 (C-10), 154.6 (C-13b), 136.8 (C-2), 136.5 (C-11), 134.2 (C-18a), 134.0 (C-9a), 119.0 (C-4), 118.8 (C-13), 112.8 (C-4a), 112.7 (C-13a), 112.2 (C-7a), 107.6 (C-3), 107.0 (C-12), 105.4 (C-16b), 81.9 (C-6), 78.5 (C-15), 61.7 (OCH_3_), 61.6 (OCH_3_), 56.3 (OCH_3_), 56.2 (OCH_3_), 33.8 (NCH_3_), 33.3 (NCH_3_), 52.3 (C-6a), 39.7 (C-19), 31.1 (C-16), 29.3 (15-CH_3_), 28.5 (6-CH_3_), 26.3 (C-16a), 25.5 (C-7), and 21.0 (6-CH_3_)

***Barbinervic acid (5)***: brown powder; ^1^H-NMR (500 MHz, methanol-*d_4_*): *δ* (ppm) = 5.30 (1H, *t*, *J* = 3.4 Hz, H-12), 3.62 (1H, *dd*, *J* = 11.4, 4.6 Hz, H-3), 3.55 (1H, *d*, *J* = 10.9 Hz, H-24b), 3.32 (1H, *d*, *J* = 10.9 Hz, H-24a), 2.52 (1H, s, H-18), 1.35 (3H, s, H-27), 1.21 (3H, s, H-29), 0.99 (3H, s, H-25), 0.94 (3H, s, H-30), 0.81 (3H, s, H-26), and 0.72 (3H, s, H-23). ^13^C-NMR (125 MHz, methanol-*d_4_*): *δ* (ppm) = 180.8 (C-28), 138.7 (C-13), 128.1 (C-12), 72.6 (C-3), 72.2 (C-19), 66.1 (C-24), 53.8 (C-18), 45.5 (C-17), 47.4 (C-5), 47.1 (C-9), 41.8 (C-4), 41.6 (C-20), 41.2 (C-14), 39.6 (C-8), 38.1 (C-1), 37.6 (C-22), 36.5 (C-10), 32.3 (C-7), 28.1 (C-15), 26.0 (C-2), 25.9 (C-21), 25.7 (C-29), 24.9 (C-16), 23.4 (C-27), 23.2 (C-11), 17.9 (C-6), 16.1 (C-26), 15.1 (C-30), 14.8 (C-25), and 11.3 (C-23)

***Quinovic acid 3β-O-α-L-rhamnopyranoside (6)***: brown powder; ^1^H-NMR (500 MHz, methanol-*d_4_*): *δ* (ppm) = 5.61 (1H, *dd,J* = 5.2, 2.5 Hz, H-12), 3.07 (1H, *dd*, *J* = 11.4, 4.8 Hz, H-3), 2.28 (2H, *m*, H-9, H-18), 1.00 (3H, s, H-25), 0.94 (3H, *s*, H-24, H-30), 0.92 (3H, *s*, H-29), 0.91 (3H, *s*, H-26), 0.81 (3H, *s*, H-23), and 0.78 (1H, *m*, H-5). L-rhamnose: 4.75 (1H, *d*, *J* = 1.6 Hz, H-1′), 3.84 (1H, *dd*, *J* = 3.2, 1.7 Hz, H-2′), 3.65 (1H, *dd*, *J* = 9.5, 3.3 Hz, H-3′), 3.35 (1H, *t*, *J* = 9.5 Hz, H-4′), 3.71 (1H, *dq*, *J* = 6.3, 9.4 Hz, H-5′), and 1.20 (3H, *d*, *J* = 6.3 Hz, H-6′). ^13^C-NMR (125 MHz, methanol-*d_4_*): *δ* (ppm) = 180.1 (C-28), 177.5 (C-27), 132.5 (C-13), 129.0 (C-12), 103.0 (C-1′), 88.9 (C-3), 72.6 (C-4′), 71.4 (C-3′), 71.1 (C-2′), 68.4 (C-5′), 55.8 (C-14), 55.3 (C-5), 54.1 (C-18), 47.9 (C-17), 46.6 (C-9), 39.3 (C-8), 38.9 (C-19), 38.6 (C-4), 38.4 (C-1), 36.8 (C-10, C-20), 36.4 (C-22), 36.2 (C-7), 29.8 (C-21), 27.3 (C-24), 25.3 (C-2, C-15), 25.1 (C-16), 22.4 (C-11), 20.1 (C-30), 18.0 (C-6), 16.6 (C-26, C-29), 17.6 (C-6′).15.4 (C-23), and 15.4 (C-25)

***Scopoletin (9)***: yellow needle; ^1^H-NMR (500 MHz, methanol-*d_4_*): *δ* (ppm) = 7.80 (1H, *d*, *J* = 9.4 Hz, H-4), 7.12 (1H, *s*, H-5), 6.80 (1H, *s*, H-8), 6.21 (1H, *d*, *J* = 9.4 Hz, H-3), and 3.91 (3H, *s*, O-CH_3_). ^13^C-NMR (125 MHz, methanol-*d_4_*): *δ* (ppm) = 162.9 (C-2), 151.4 (C-8a), 150.0 (C-7), 145.6 (C-6), 144.7 (C-4), 111.3 (C-4a), 111.2 (C-3), 108.4 (C-5), 102.7 (C-8), and 55.6 (O-CH_3_)

***Gallic acid (10)***: white amorphous powder; ^1^H-NMR (500 MHz, methanol-*d_4_*): *δ* (ppm) = 7.07 (2H, *s*, H-2/H-6). ^13^C-NMR (125 MHz, methanol-*d_4_*): *δ* (ppm) = 168.9 (C = 0), 145.3 (C-3), 138.2 (C-4), 120.5 (C-1), and 108.8 (C-2/C-6)

***Quercetin 3-O-β-D-glucopyranoside (11)***: yellow amorphous powder; ^1^H-NMR (500 MHz, methanol-*d_4_*): *δ* (ppm) = 7.72 (1H, *d*, *J* = 2.1 Hz, H-2′),7.61 (1H, *dd*, *J* = 8.4, 2.1 Hz, H-6′), 6.89 (1H, *d*, *J* = 8.4 Hz, H-5′), 6.43 (1H, *d*, *J* = 2.1 Hz, H-8), 6.23 (1H, *d*, *J* = 2.1 Hz, H-6), 5.27 (1H, *d*, *J* = 7.6 Hz, H-1^″^), 3.72 (1H, *m*, H-6a^″^), 3.58 (1H, *m*, H-6b^″^), 3.50 (1H, *m*, H-2a^″^), 3.44 (1H, m, H-3^″^), 3.37 (1H, *m*, H-4^″^), and 3.28 (1H, m, H-5^″^). ^13^C-NMR (125 MHz, methanol-*d_4_*): *δ* (ppm) = 179.1 (C-4), 165.7 (C-7), 162.7 (C-5), 161.5 (C-2), 158.5 (C-9), 149.2 (C-4′), 145.6 (C-3′), 135.3 (C-3), 122.4 (C-1′), 122.8 (C-6′), 116.9 (C-2′), 116.5 (C-5′), 105.3 (C-10), 103.8 (C-1^″^), 99.3 (C-6), 94.2 (C-8), 78.0 (C-5^″^), 77.6 (C-3^″^), 62.3 (C-6^″^), 75.2 (C-2^″^), and 70.7 (C-4^″^)

***Kaempferol 3-O-α-L-rhamnopyranoside-7-O-α-L-rhamnopyranoside (12)***: yellow amorphous powder; ^1^H-NMR (500 MHz, CDCl_3_+methanol-*d_4_*): *δ* (ppm) = 7.77 (1H, *d*, *J* = 8.8 Hz, H-2′/H-6′), 6.94 (1H, *d*, *J* = 8.8 Hz, H-3′/H-5′), 6.70 (1H, *d*, *J* = 2.1 Hz, H-8), 6.45 (1H, *d*, *J* = 2.1 Hz, H-6), 5.54 (1H, *d*, *J* = 1.2 Hz, H-1^″^), 5.40 (1H, *d*, *J* = 1.5 Hz, H-1^‴^), 4.26 (1H, *dd*, *J* = 3.2, 1.6 Hz, H-2^‴^), 4.05 (1H, *dd*, *J* = 3.3, 1.7 Hz, H-2^″^), 3.86 (1H, *dd*, *J* = 9.5, 3.4 Hz, H-2^″^), 3.74 (1H, *m*, H-3^‴^), 3.62 (1H, *dq*, *J* = 12.3, 6.1 Hz, H-5^″^), 3.50 (1H, *d*, *J* = 9.5 Hz, H-4^″^), 3.34 (1H, *m*, H-4^‴^), 3.33 (1H, *m*, H-5^‴^), 1.28 (3H, *d*, *J* = 6.2 Hz, H-6^″^), and 0.94 (3H, *d*, *J* = 5.6 Hz, H-6^‴^). ^13^C-NMR (125 MHz, CDCl_3_+methanol-*d_4_*): *δ* (ppm) = 178.5 (C-4), 162.0 (C-7), 161.5 (C-5), 160.1 (C-4′), 158.5 (C-2), 156.8 (C-9), 135.2 (C-3), 130.7 (C-2′/C-6′), 121.1 (C-1′), 115.4 (C-3′/C-5′), 106.5 (C-10), 102.0 (C-1^″^), 99.5 (C-6), 98.3 (C-1^‴^), 94.4 (C-8), 72.3 (C-4^′″^), 71.9 (C-4^″^), 70.8 (C-3^‴^), 70.4 (C-2^″^), 70.6 (C-4^‴^), 70.2 (C-2^″′^), 70.6 (C-5^″^), 69.8 (C-5^′″^), 17.1 (C-6^″′^), and 16.6 (C-6^″^)

***Borreriagenin (13)***: yellow oil; ^1^H-NMR (500 MHz, methanol-*d_4_*): *δ* (ppm) = 5.85 (1H, *d*, *J* = 1.7 Hz, H-7), 5.41 (1H, *d*, *J* = 7.6 Hz, H-6), 4.20 (1H, *m*, H-10a), 3.92 (1H, *dd*, *J* = 10.8, 4.7 Hz, H-3a), 3.87 (1H, *dd*, *J* = 10.9, 4.7 Hz, H-3b), 3.77 (1H, *m*, H-10b), 3.59 (1H, *dd*, *J* = 11.2, 4.9 Hz, H-1a), 3.52 (1H, *dd*, *J* = 10.8, 4.7 Hz, H-1b), 3.34 (1H, *m*, H-5), 3.11 (1H, *m*, H-9), and 2.97 (1H, *m*, H-4). ^13^C-NMR (125 MHz, methanol-*d_4_*): *δ* (ppm) = 179.6 (C-11), 151.9 (C-8), 123.6 (C-7), 86.7 (C-6), 63.0 (C-1), 61.4 (C-3), 53.9 (C-10), 48.6 (C-9), 44.6 (C-4), and 42.6 (C-5)

### 3.2. Antibacterial Activity

The results of *in vitro* activities of the EtOH, *n*-BuOH, and EtOAc extracts as well as some isolated compounds against pathogenic bacteria are presented in Table 1. The *n*-BuOH and EtOAc extracts showed antibacterial activity against Gram-positive and Gram-negative bacteria (MIC = 32–64 *μ*g/mL; MBC = 64–256 *μ*g/mL) whereas the EtOH extract was active only on Gram-negative bacteria (MIC = 32–128 *μ*g/mL; MBC = 64–256 *μ*g/mL). The antibacterial activity of the plant extracts can be classified as significant (MIC < 100 *μ*g/mL), moderate (100 < MIC ≤ 625 *μ*g/mL), and weak (MIC > 625 *μ*g/mL) [24]. According to this classification, the inhibition potential of the tested extracts could be considered as significant to moderate. The *n*-BuOH extract was the most active with a lowest MIC value of 32 *μ*g/mL against *Pseudomonas aeruginosa* ATCC 76110, *Staphylococcus aureus* ATCC 25923, and *Klebsiella pneumoniae* 22 and of 64 *μ*g/mL against *Escherichia coli* ATCC 25922 followed by the EtOH extract which displayed a MIC value of 32 *μ*g/mL on *K. pneumoniae* 22 and of 64 *μ*g/mL on *P. aeruginosa* ATCC 76110, *S. aureus* ATCC 25923, and *K. pneumoniae* 22. The isolated secondary metabolites showed inhibition ranging from moderate to weak according to the scale which states that antimicrobial activity of pure compounds can be classified as significant (MIC < 10 *μ*g/mL), moderate (10 < MIC ≤ 100 *μ*g/mL), and weak (MIC > 100 *μ*g/mL) [24]. Scopoletin (**9**) exhibited a moderate activity with a MIC value of 16 *μ*g/mL against *S. aureus* ATCC 25923, *K. pneumoniae* 22, and a MIC value of 32 *μ*g/mL against *P. aeruginosa* ATCC 76110 while barbinervic acid (**5**) showed a moderate activity against *P. aeruginosa* ATCC 76110 and *K. pneumoniae* 22 with a MIC value of 32 *μ*g/mL.

## 4. Discussion

### 4.1. Chemotaxonomy

The present study reports the first phytochemical investigation of *P. succulenta* which led to the isolation and structure elucidation of thirteen compounds including four alkaloids (veprisine (**1**), naucleofficine III (**2**), vepridimerine B (**3**), and vepridimerine C (**4**)), three triterpenes (barbinervic acid (**5**), 3-*O*-*α*-L-rhamnopyranosyl quinovic acid (**6**), and oleanolic acid (**7**)), one steroid (*β*-sitosterol-3-*O*-*β*-D-glucopyranoside (**8**)), four phenolic compounds (scopoletin (**9**), gallic acid (**10**), quercetin-3-*O*-*β*-glucopyranoside (**11**), and kaempferol 3-*O*-*α*-L-rhamnopyranoside-7-*O*-*α*-L-rhamnopyranoside (**12**)), and one iridoid (borreriagenin (**13**)). Although all these compounds are isolated from *P. succulenta* for the first time, some of them have already been obtained from other *Psychotria* species. It is the case of barbinervic acid (**5**) previously found in *P. stachyoides* [25], oleanolic acid (**7**) and *β*-sitosterol-3-*O*-*β*-D-glucopyranoside (**8**) isolated from *P. viridis* [26], and scopoletin (**9**) obtained from *P. vellosiana* and *P. stachyoides* [25, 27]. The results obtained are in agreement with the chemotaxonomy of plants of the genus *Psychotria* since according to Calixto et al. (2016), they are characterized as an abundant source of indole, monoterpene indole, quinoline, and isoquinoline alkaloids as well as flavonoids. Furthermore, approximately 52% of the metabolites reported were characterized as alkaloids, followed by triterpenes (12%) and flavonoids (6%) along with constituents of other classes [28]. The monoterpene indole alkaloid naucleofficine III (**2**) isolated during our investigation has already been obtained from the stems of *Nauclea officinalis* [13] also belonging to the family Rubiaceae. Nevertheless, the isolation from *P. succulenta* of a metabolite belonging to this class of compounds reported to derive biosynthetically from the coupling of tryptophan, and the iridoid seccolaganin is not surprising since many congeners have been isolated from other *Psychotria* species [28–30]. Furthermore, regarding the distribution of the major secondary metabolites in Rubiaceae, indole alkaloids are indicated as the main chemical markers of this family [31].

Veprisine (**1**), vepridimerine B (**3**), and vepridimerine C (**4**) are quinolone-terpene alkaloids occurring mainly in plants of the Rutaceae family [12, 14, 32, 33]. To the best of our knowledge, this is the first report of their isolation from Rubiaceae. Nevertheless, the cooccurrence of indole and quinoline alkaloids in the same plant species is well documented. These two classes of alkaloids were obtained from *Araliopsis soyauxii* (Rutaceae) [33], *Melodinus yunnanensis* (Apocynaceae) [34], *Alstonia scholaris* (Apocynaceae) [35], and *Clausena lansium* (Rutaceae) [36]. This further confirmed the fact that biosynthetically, quinoline alkaloids may be derived from ring expansion of indole alkaloids [35]. This seems to be the first report on the isolation of the flavonoid glycosides quercetin-3-*O*-*β*-glucopyranoside (**11**) and kaempferol 3-*O*-*α*-L-rhamnopyranoside-7-*O*-*α*-L-rhamnopyranoside (**12**) from a plant of the *Psychotria* genus although these secondary metabolites have already been found in the family Rubiaceae, precisely in *Hedyotis diffusa* and *Hedyotis verticillata*, respectively [31]. The iridoid borreriagenin (**13**) obtained during this work has already been isolated from some plants of the Rubiaceae family including *Borreria verticillata* [21] and *Morinda longifolia* [37], but to the best of our knowledge, this is the first report of its isolation from a plant of the genus *Psychotria*.

### 4.2. Antibacterial Activity

The findings of the present study showed differences between the antibacterial activities of extracts from *P. succulenta* seeds. This suggests that *P. succulenta* contains several active principles with different polarities as shown by the nature of the isolated compounds. Indeed, the antibacterial activities of medicinal plants are correlated with the presence in their extracts of one or more bioactive secondary metabolites [38]. The n-BuOH extract was the most active following in decreasing order by the EtOAc extract and MeOH extract. This result reinforces the concept that *P. succulenta* contains also polar antibacterial compounds. These differences in antibacterial activities from different solvents had also been observed [9, 11]. Hence, the n-BuOH extract was expected to produce significant active principles in this research. However, the results showed that ethyl acetate was the better solvent compared to the n-BuOH to isolate the phytochemicals (compounds **2**, **5**, and **9**) that are most active toward the tested bacteria from *P. succulenta*. The ethyl acetate is a semipolar solvent and could effectively extract active principles with semipolar properties such as alkaloids, sterols, terpenoids, flavonoids, and glycosides from the plant [39].

Different parts (leaves, roots, barks, and rhizomes) of plants of the genus *Psychotria* are commonly used in traditional medicines for treating bronchial and gastrointestinal disorders such as cough, bronchitis, ulcer, and stomachache [2, 40]. They are also used to cure infections of the female reproductive system [3]. Previous pharmacological works carried out on other *Psychotria* species like *P. microlabastra* (leaves, stem, and roots bark), *P. gardineri* (branches and leaves), and *P. nigra* (branches and leaves) have shown that methanol, dichloromethane, and hexane extracts exhibit antibacterial activities [41]. Our results allow us not only to validate the use of *P. succulenta* in traditional medicine but also to approve the literature data.

The findings of the present study showed that the MBC values are in general fourfold lesser than the MIC values on the corresponding bacteria; suggesting that the extracts and some isolated compounds from *P. succulenta* seeds have a bactericidal effect on the sensitive bacteria [42].

The results of the antibacterial activity of some isolated compound from *P. succulenta* seeds are in agreement with those of the literature. Indeed, veprisine isolated from the root wood of *Teclea maniensis* (Rutaceae) exhibited moderate to higher antimycobacterial activity against two mycobacterial strains, namely, *Mycobacterium madagascariense* DSM 44641 and *Mycobacterium indicus pranii* DSM 45239 with the MIC values of 657.9 *μ*M and 2.63 × 10^3^ *μ*M, respectively [12]. Oleanolic acid isolated from *Miconia* species displayed antibacterial effect with MIC values ranging from 30 *μ*g/mL to 70 *μ*g/mL [43]. A phenolic coumarin scopoletin (7-hydroxy-6-methoxycoumarin) from *Lasianthus lucidus* Blume (Rubiaceae) proved to be effective against *Pseudomonas aeruginosa* ATCC 27853 (AmpC *β*-lactamase producing strain) and *P. aeruginosa* DMSC 37166 [44]. It was found that gallic acid had antimicrobial activity against *P. aeruginosa*, *E. coli*, *S. aureus*, and *Lysteria monocytogenes* through hydrophobicity changes, decrease of negative surface charge, and occurrence of local rupture or pore formation in the cell membranes with consequent leakage of essential intracellular constituents [45]. Finally, 10-acetyl borreriagenin isolated from the aerial parts of *Hedyotis pilulifera* (Rubiaceae) showed antibacterial activity against *Staphylococcus aureus*, with an MIC value of 100 *μ*g/mL [46]. To the best of our knowledge, this is the first report on the antibacterial activities of the extracts, naucleofficine III, vepridimerine B, vepridimerine C, barbinervic acid, 3-*O*-*α*-L-rhamnopyranosyl quinovic acid, quercetin-3-*O*-*β*-D-glucopyranoside, kaempferol 3-*O*-*α*-L-rhamnopyranoside-7-*O*-*α*-L-rhamnopyranoside, and borreriagenin from *P. succulenta* seeds. The overall study emphasizes the potential of *P. succulenta* seeds as a sustainable source of broad spectrum antibacterial agents.

## 5. Conclusion

In conclusion, the phytochemical investigation of the seeds of *P. succulenta* led to isolation and characterization of thirteen compounds, namely, veprisine (1), naucleofficine III (**2**), vepridimerine B (**3**), vepridimerine C (**4**), barbinervic acid (**5**), quinovic acid 3*β*-*O*-*α*-L-rhamnoside (**6**), oleanolic acid (**7**), *β*-sitosterol-3-*O*-*β*-D-glucopyranoside (**8**), scopoletin (**9**), gallic acid (**10**), quercetin 3-*O*-*β*-D-glucopyranoside (**11**), kaempferol 3-*O*-*α*-L-rhamnopyranoside-7-*O*-*α*-L-rhamnopyranoside (**12**), and borreriagenin (**13**). All these secondary metabolites were isolated from this plant species for the first time although some of them have already been isolated from plants of the genus *Psychotria*. Our results clearly showed that *P. succulenta* has a close chemotaxonomic relationship with other plants of the genus *Psychotria*. Furthermore, the extracts and some isolated compounds showed antibacterial activity against pathogenic bacteria, confirming the use of *P. succulenta* in traditional medicine to cure infectious diseases. Barbinervic acid (**5**) and scopoletin (**9**) were the most antibacterial principles of *P. succulenta*.

## Figures and Tables

**Table 1:** Antibacterial activity of the extracts and some isolated compounds from *P. succulenta* seeds.

Samples	Parameters	Bacterial species			
		*Pseudomonas aeruginosa*	*Staphylococcus aureus*	*Escherichia coli*	*Klebsiella pneumoniae*
EtOH extract	MIC/MBC	128/256	-/-	64/128	32/64
EtOAc extract	MIC/MBC	64/256	64/128	-/-	64/128
*n*-BuOH extract	MIC/MBC	32/64	32/128	64/128	32/128
1	MIC/MBC	128/128	-/-	32/128	128/128
2	MIC/MBC	64/64	128/-	128/128	32/64
3	MIC/MBC	64/128	64/128	-/-	128/128
4	MIC/MBC	64/-	64/-	-/-	-/-
5	MIC/MBC	64/128	32/64	64/64	32/64
6	MIC/MBC	128/-	-/-	-/-	128/-
9	MIC/MBC	32/64	16/32	64/128	16/32
10	MIC/MBC	128/-	-/-	-/-	64/-
11	MIC/MBC	128/-	128/-	-/-	128/-
12	MIC/MBC	64/-	-/-	-/-	32/-
13	MIC/MBC	128/-	128/-	64/-	-/-
Ciprofloxacine	MIC/MBC	2/4	8/16	8/16	8/16

MIC: minimum inhibitory concentration; MBC: minimum bactericidal concentration; MIC and MBC in *μ*g/mL; -: not active at concentration up to 256 *μ*g/mL for the compounds and 2048 *μ*g/mL for the extracts.

## Data Availability

The datasets generated and analyzed during the current study are available from the corresponding author upon reasonable request.

## Abbreviations

^13^C-NMR: Carbon thirteen nuclear magnetic resonance
^1^H-NMR: Proton nuclear magnetic resonance
2D NMR: Two-dimension nuclear magnetic resonance
ATCC: American Type Culture Collection
CC: Column chromatography
CFU: Colony forming unit
COSY: Correlation spectroscopy
DMSO: Dimethylsulfoxide
EtOAc: Ethyl acetate
HMBC: Heteronuclear multiple bond connectivities
HNC: Herbier National du Cameroun
HSQC: The heteronuclear single-quantum coherence
INT: *p*-Iodonitrotetrazolium
IR: Infrared
MBC: Minimum bactericidal concentration
MDR: Multidrug-resistant
MeOH: Methanol
MHA: Mueller Hinton agar
MHB: Mueller Hinton broth
MBC: Minimum bactericidal concentration
MIC: Minimum inhibitory concentration
NA: Nutrient agar
*n*-BuOH: *n*-Butanol
NMR: Nuclear magnetic resonance
Rf: Retention factor
TLC: Thin layer chromatography
TMS: Tetramethylsilane
UV: Ultraviolet.

## Supplementary Materials

Supplementary Materials Figure S1: ^1^H NMR spectrum of compound **1**. Figure S2: ^13^C NMR spectrum of compound **1**. Figure S3: ^1^H NMR spectrum of compound **2**. Figure S4: ^13^C NMR spectrum of compound **2**. Figure S5: ^1^H NMR spectrum of compound **3**. Figure S6: ^13^C NMR spectrum of compound **3**. Figure S7: ^1^H-^1^H COSY spectrum of compound **3**. Figure S8: HSQC spectrum of compound **3**. Figure S9: HMBC spectrum of compound **3**. Figure S10: NOESY spectrum of compound **3**. Figure S11: ^1^H NMR spectrum of compound **4**. Figure S12: ^13^C NMR spectrum of compound **4**. Figure S13: ^1^H-^1^H COSY spectrum of compound **4**. Figure S14: HSQC spectrum of compound **4**. Figure S15: HMBC spectrum of compound **4**. Figure S16: NOESY spectrum of compound **4**. Figure S17: ^1^H NMR spectrum of compound **5**. Figure S18: ^13^C NMR spectrum of compound **5**. Figure S19: ^1^H NMR spectrum of compound **6**. Figure S20: ^13^C NMR spectrum of compound **6**. Figure S21: ^1^H NMR spectrum of compound **9**. Figure S22: ^13^C NMR spectrum of compound **9**. Figure S23: ^1^H NMR spectrum of compound **10**. Figure S24: ^13^C NMR spectrum of compound **10**. Figure S25: ^1^H NMR spectrum of compound **11**. Figure S26: ^13^C NMR spectrum of compound **11**. Figure S27: ^1^H NMR spectrum of compound **12**. Figure S28: ^13^C NMR spectrum of compound **12**. Figure S29: ^1^H NMR spectrum of compound **13**. Figure S30: ^13^C NMR spectrum of compound **13**. Figure S31: Microtiter plate images after INT colorimetric assay.
