# Supplementary material for: Chemotaxonomy and Antibacterial Activity of the Extracts and Chemical Constituents of Psychotria succulenta Hiern. (Rubiaceae)
Source: Biomed Res Int. 2022 Jun 15;2022:7856305. doi: 10.1155/2022/7856305 (PMC9217553; doi:10.1155/2022/7856305)
Supplement: Supplementary Materials — Figure S1: 1H NMR spectrum of compound 1. Figure S2: 13C NMR spectrum of compound 1. Figure S3: 1H NMR spectrum of compound 2. Figure S4: 13C NMR spectrum of compound 2. Figure S5: 1H NMR spectrum of compound 3. Figure S6: 13C NMR spectrum of compound 3. Figure S7: 1H-1H COSY spectrum of compound 3. Figure S8: HSQC spectrum of compound 3. Figure S9: HMBC spectrum of compound 3. Figure S10: NOESY spectrum of compound 3. Figure S11: 1H NMR spectrum of compound 4. Figure S12: 13C NMR spectrum of compound 4. Figure S13: 1H-1H COSY spectrum of compound 4. Figure S14: HSQC spectrum of compound 4. Figure S15: HMBC spectrum of compound 4. Figure S16: NOESY spectrum of compound 4. Figure S17: 1H NMR spectrum of compound 5. Figure S18: 13C NMR spectrum of compound 5. Figure S19: 1H NMR spectrum of compound 6. Figure S20: 13C NMR spectrum of compound 6. Figure S21: 1H NMR spectrum of compound 9. Figure S22: 13C NMR spectrum of compound 9. Figure S23: 1H NMR spectrum of compound 10. Figure S24: 13C NMR spectrum of compound 10. Figure S25: 1H NMR spectrum of compound 11. Figure S26: 13C NMR spectrum of compound 11. Figure S27: 1H NMR spectrum of compound 12. Figure S28: 13C NMR spectrum of compound 12. Figure S29: 1H NMR spectrum of compound 13. Figure S30: 13C NMR spectrum of compound 13. Figure S31: Microtiter plate images after INT colorimetric assay. [file 7856305.f1.pdf]

Chemotaxonomy and antibacterial activity of the extracts and chemical constituents of *Psychotria succulenta* Hiern. (Rubiaceae)

Darille Claudia Ngnokam Jouogo[1], Jean-De-Dieu Tamokou[2,*], Rémy Bertrand Teponno[1,*], Germaine Matsuete Takongmo[2], Laurence Voutquenne-Nazabadioko[3], Léon Azefack Tapondjou[1], David Ngnokam[1]

[1]Research Unit of Applied and Environmental Chemistry, Department of Chemistry, Faculty of Science, University of Dschang, P.O. Box 67. Dschang Cameroon

[2]Research Unit of Microbiology and Antimicrobial Substances, Department of Biochemistry, Faculty of Science, University of Dschang, P.O. Box 67. Dschang Cameroon

[3]*Groupe Isolement et Structure, Institut de Chimie Moléculaire de Reims (ICMR), CNRS UMR 7312, Bat. 18 B.P. 1039, 51687 Reims Cedex 2, France*

Supplementary Material

FIGURE captions

FIGURE S1: ^{1}H NMR spectrum of compound **1**

FIGURE S2: ^{13}C NMR spectrum of compound **1**

FIGURE S3: ^{1}H NMR spectrum of compound **2**

FIGURE S4: ^{13}C NMR spectrum of compound **2**

FIGURE S5: ^{1}H NMR spectrum of compound **3**

FIGURE S6: ^{13}C NMR spectrum of compound **3**

FIGURE S7: ^{1}H-^{1}H COSY spectrum of compound **3**

FIGURE S8: HSQC spectrum of compound **3**

FIGURE S9**:** HMBC spectrum of compound **3**

FIGURE S10**:** NOESY spectrum of compound **3**

FIGURE S11: ^{1}H NMR spectrum of compound **4**

FIGURE S12: ^{13}C NMR spectrum of compound **4**

FIGURE S13: ^{1}H-^{1}H COSY spectrum of compound **4**

FIGURE S14: HSQC spectrum of compound **4**

FIGURE S15: HMBC spectrum of compound **4**

FIGURE S16: NOESY spectrum of compound **4**

FIGURE S17: ^{1}H NMR spectrum of compound **5**

FIGURE S18: ^{13}C NMR spectrum of compound **5**

FIGURE S19: ^{1}H NMR spectrum of compound **6**

FIGURE S20: ^{13}C NMR spectrum of compound **6**

FIGURE S21: ^{1}H NMR spectrum of compound **9**

FIGURE S22: ^{13}C NMR spectrum of compound **9**

FIGURE S23: ^{1}H NMR spectrum of compound **10**

FIGURE S24: ^{13}C NMR spectrum of compound **10**

FIGURE S25: ^{1}H NMR spectrum of compound **11**

FIGURE S26: ^{13}C NMR spectrum of compound **11**

FIGURE S27: ^{1}H NMR spectrum of compound **12**

FIGURE S28: ^{13}C NMR spectrum of compound **12**

FIGURE S29: ^{1}H NMR spectrum of compound **13**

FIGURE S30: ^{13}C NMR spectrum of compound 1**3**

FIGURE S31: Microtiter plate images after INT colorimetric assay.

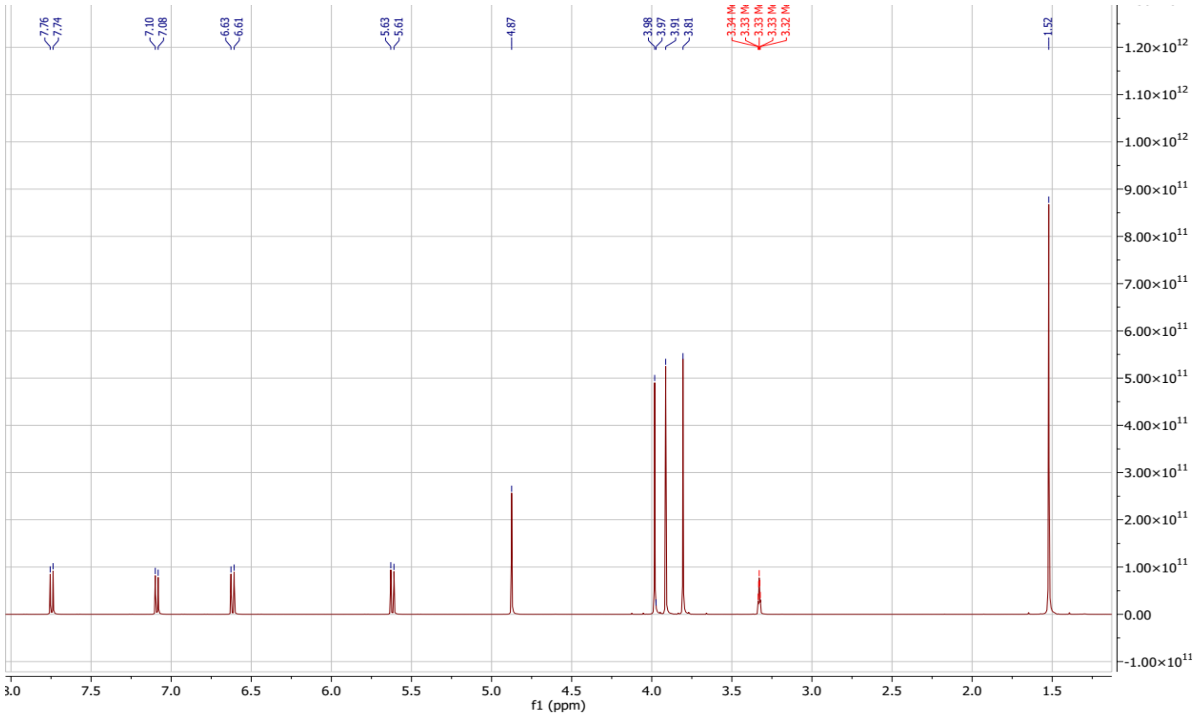

FIGURE S1: ^{1}H NMR spectrum of compound **1**

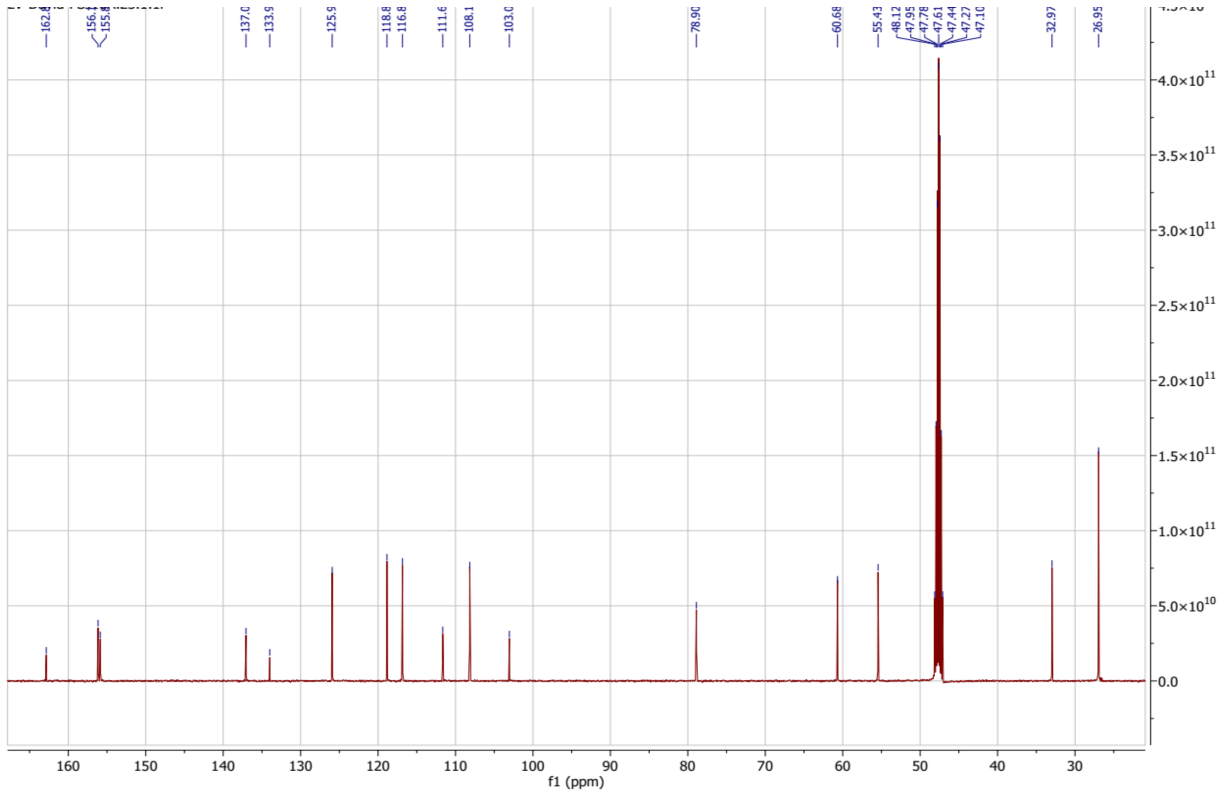

FIGURE S2: ^{13}C NMR spectrum of compound **1**

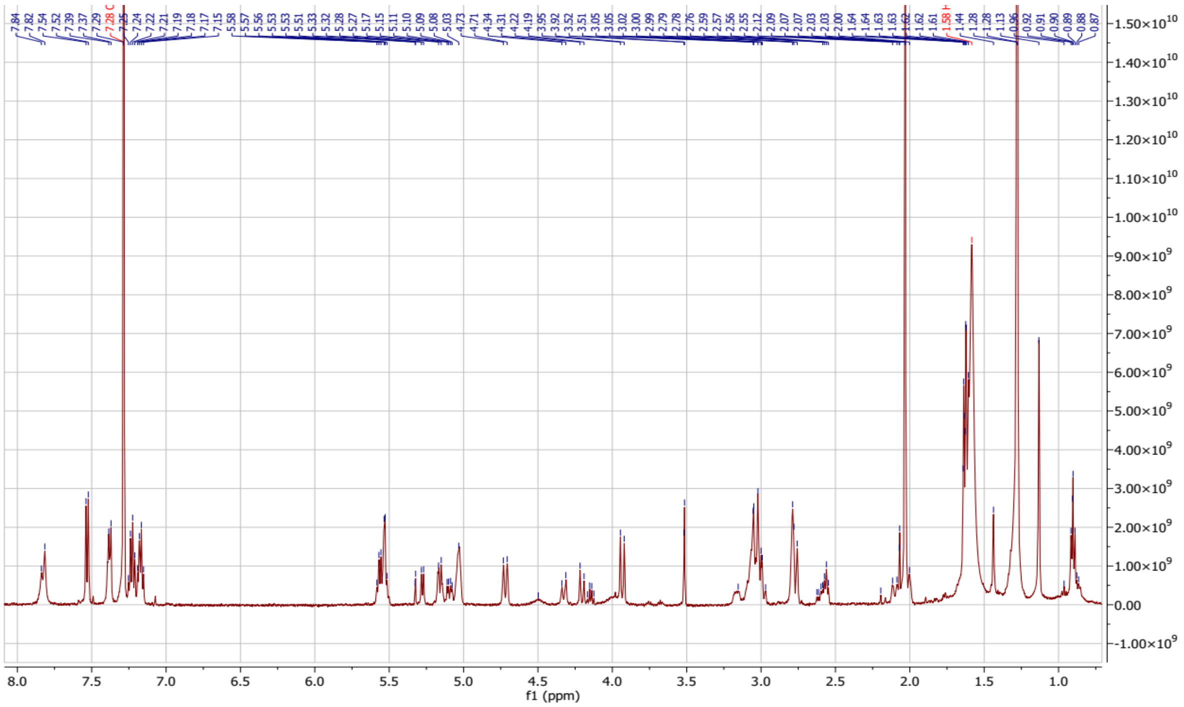

FIGURE S3: ^{1}H NMR spectrum of compound **2**

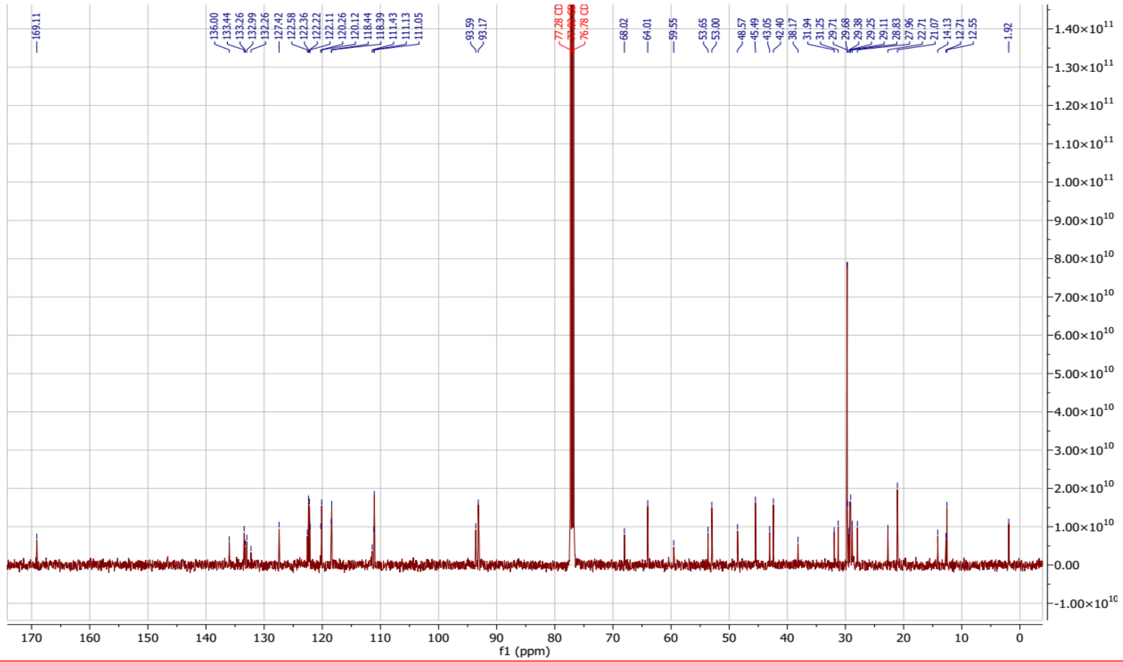

FIGURE S4: ^{13}C NMR spectrum of compound **2**

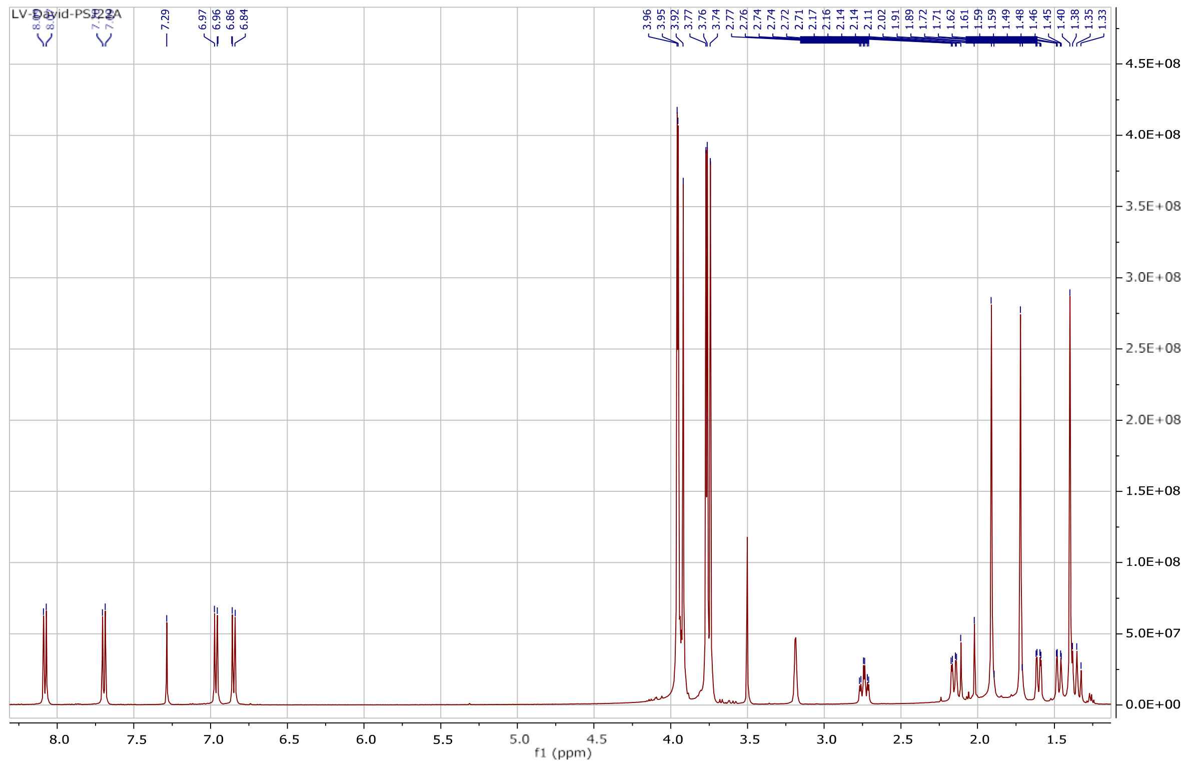

FIGURE S5: ^{1}H NMR spectrum of compound **3**

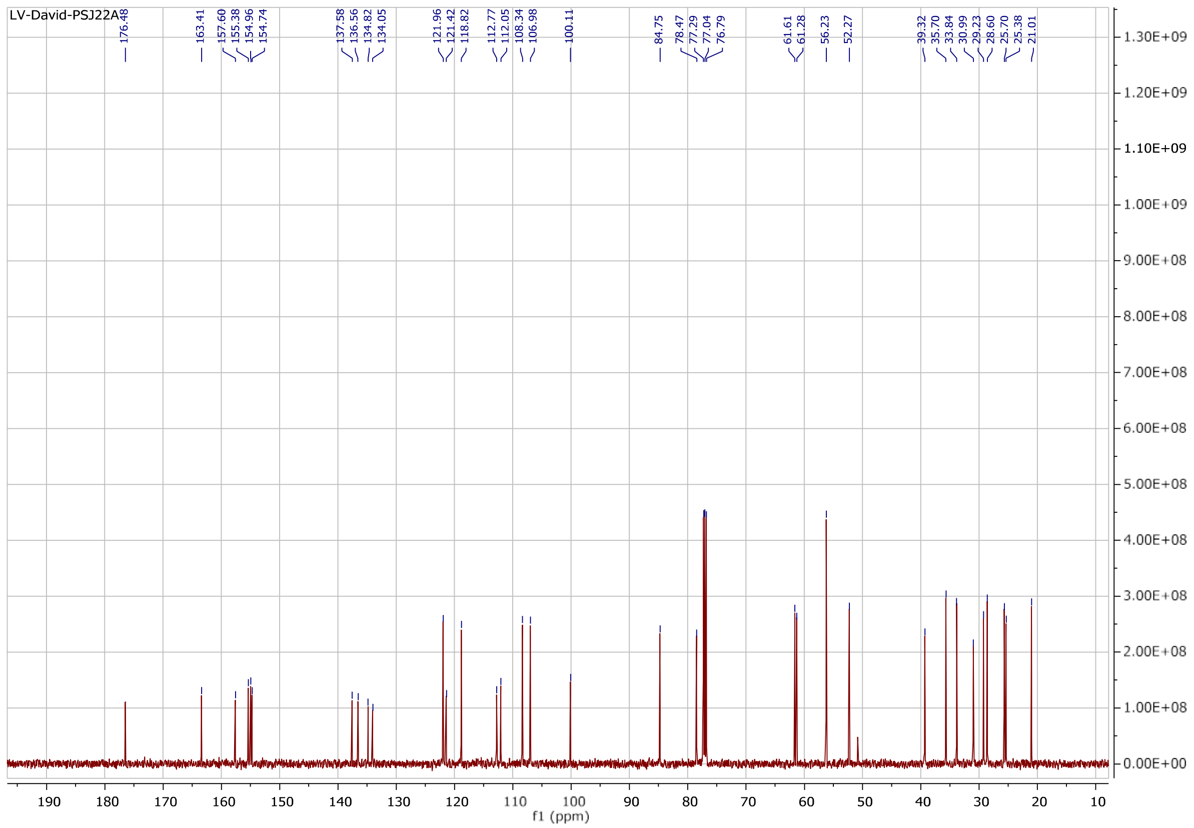

FIGURE S6: ^{13}C NMR spectrum of compound **3**

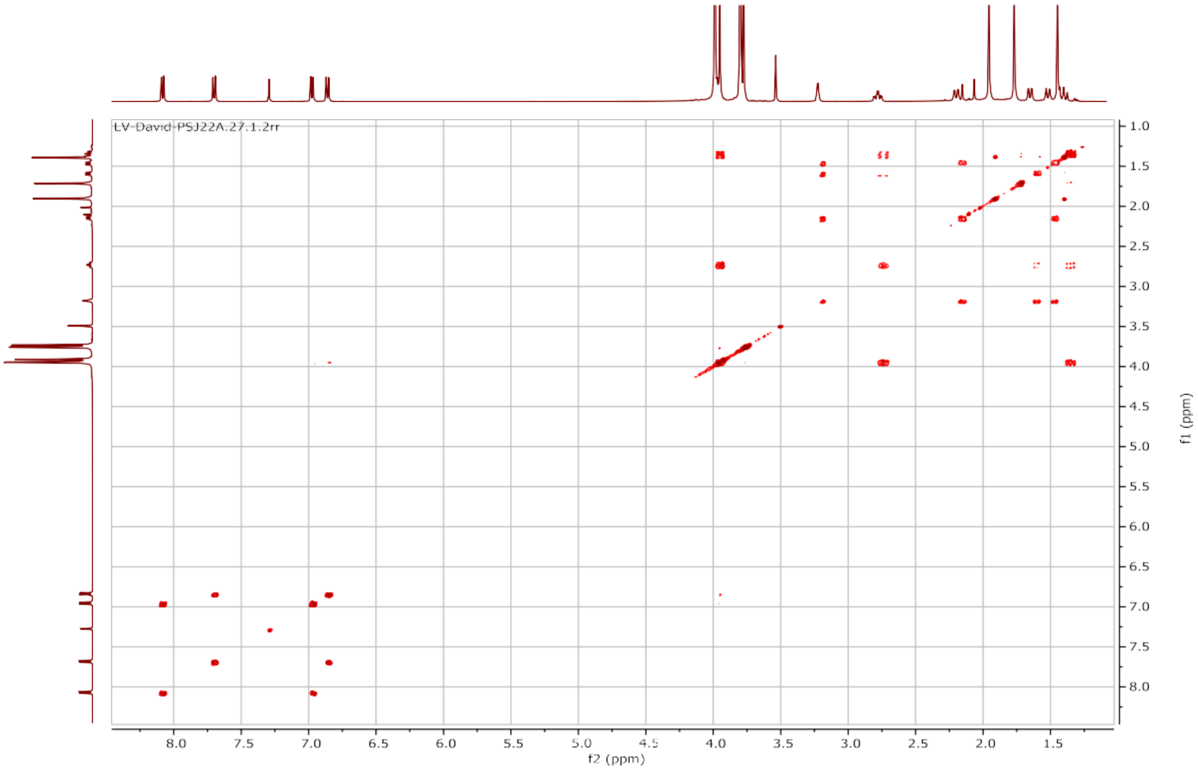

FIGURE S7: ^{1}H-^{1}H COSY spectrum of compound **3**

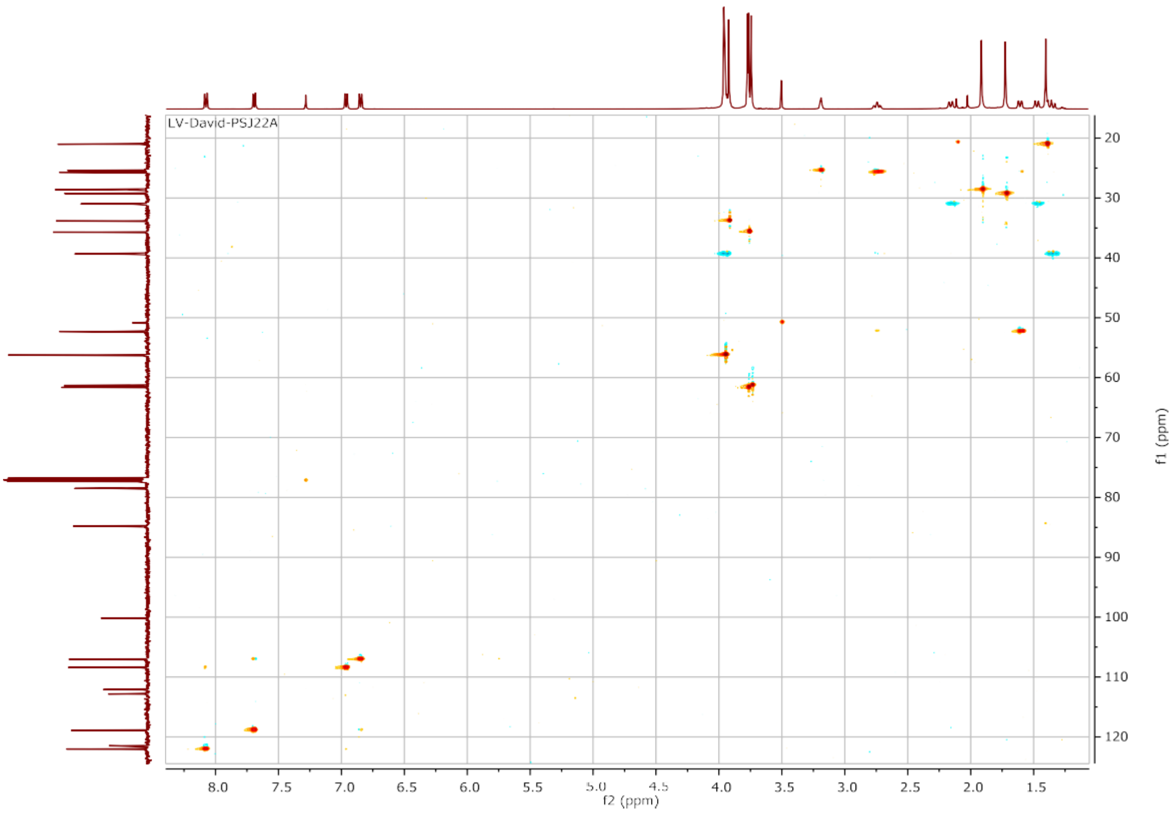

FIGURE S8: HSQC spectrum of compound **3**

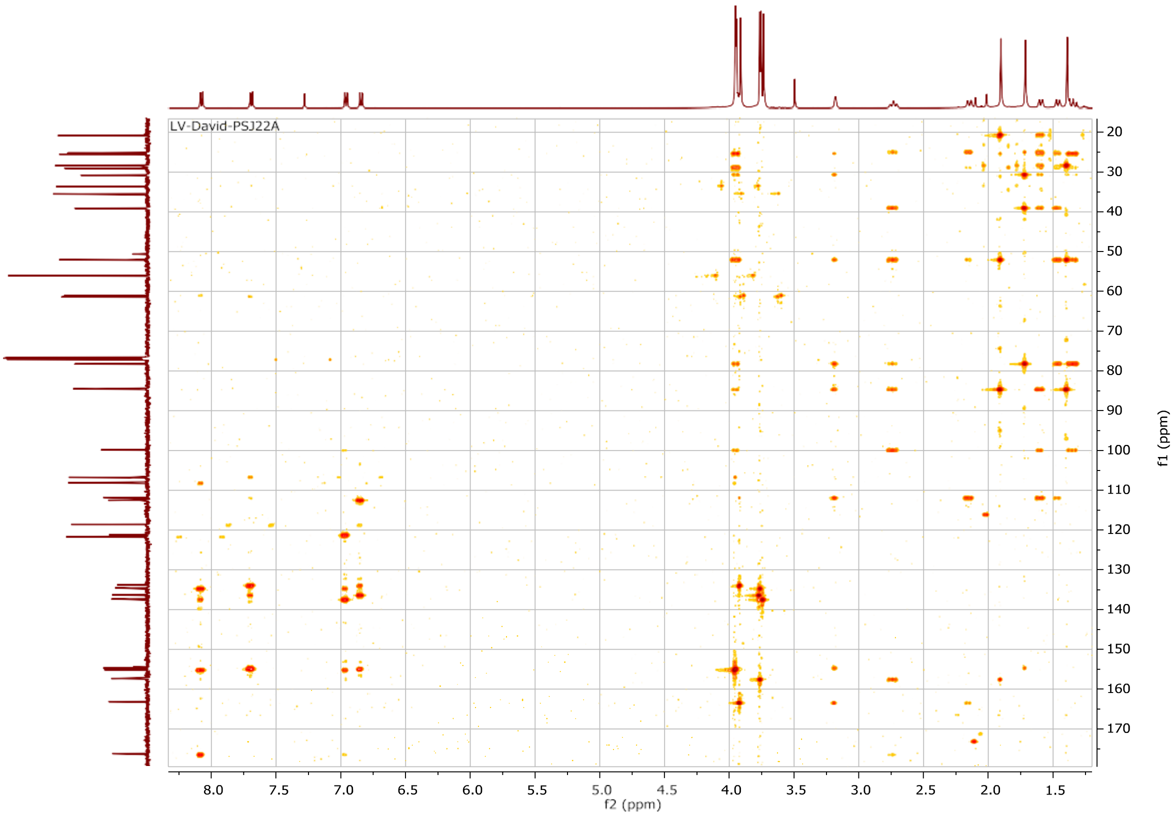

FIGURE S9: HMBC spectrum of compound **3**

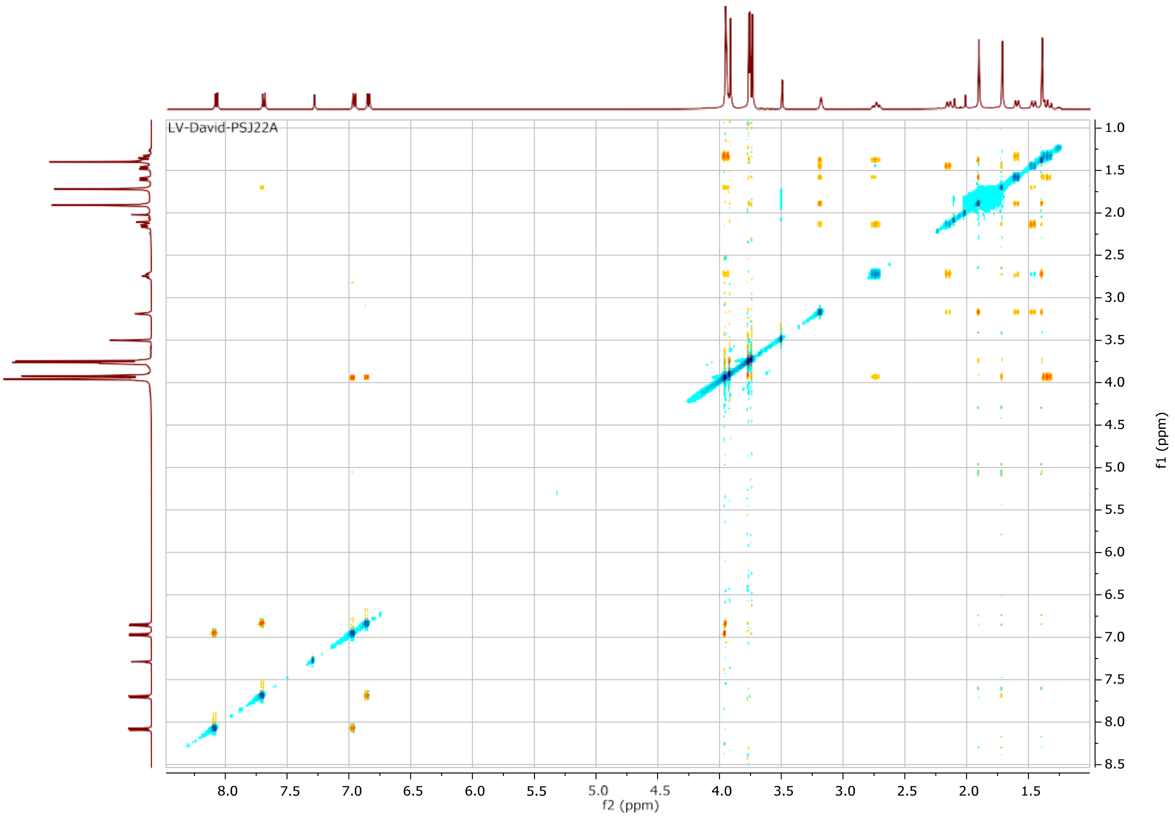

FIGURE S10: NOESY spectrum of compound **3**

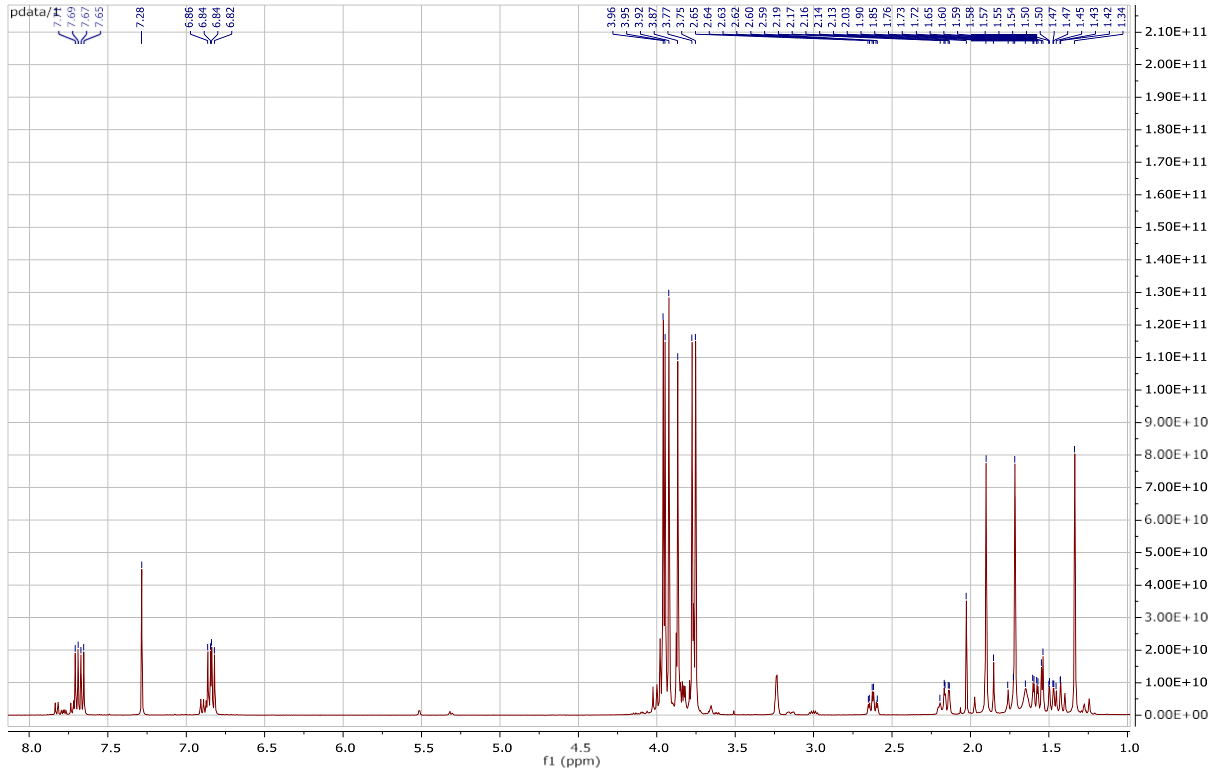

FIGURE S11: ^{1}H NMR spectrum of compound **4**

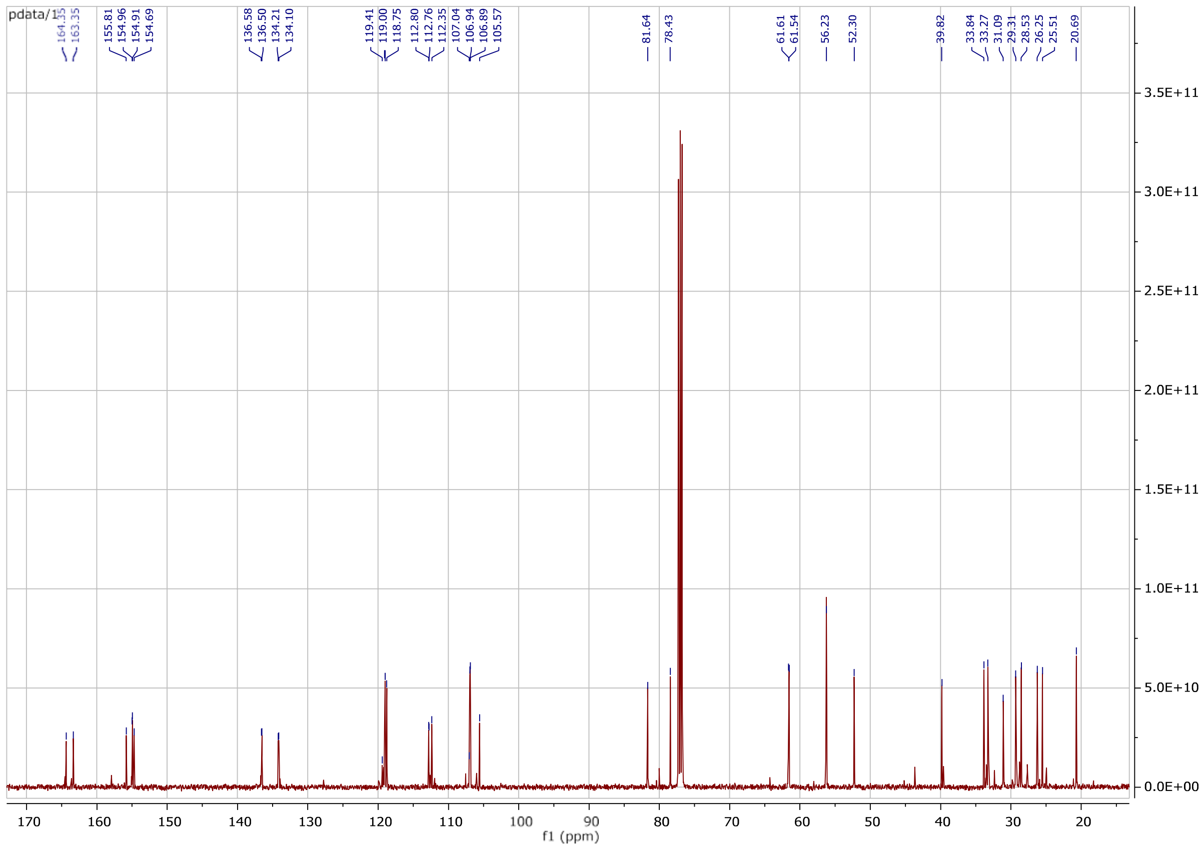

FIGURE S12: ^{13}C NMR spectrum of compound **4**

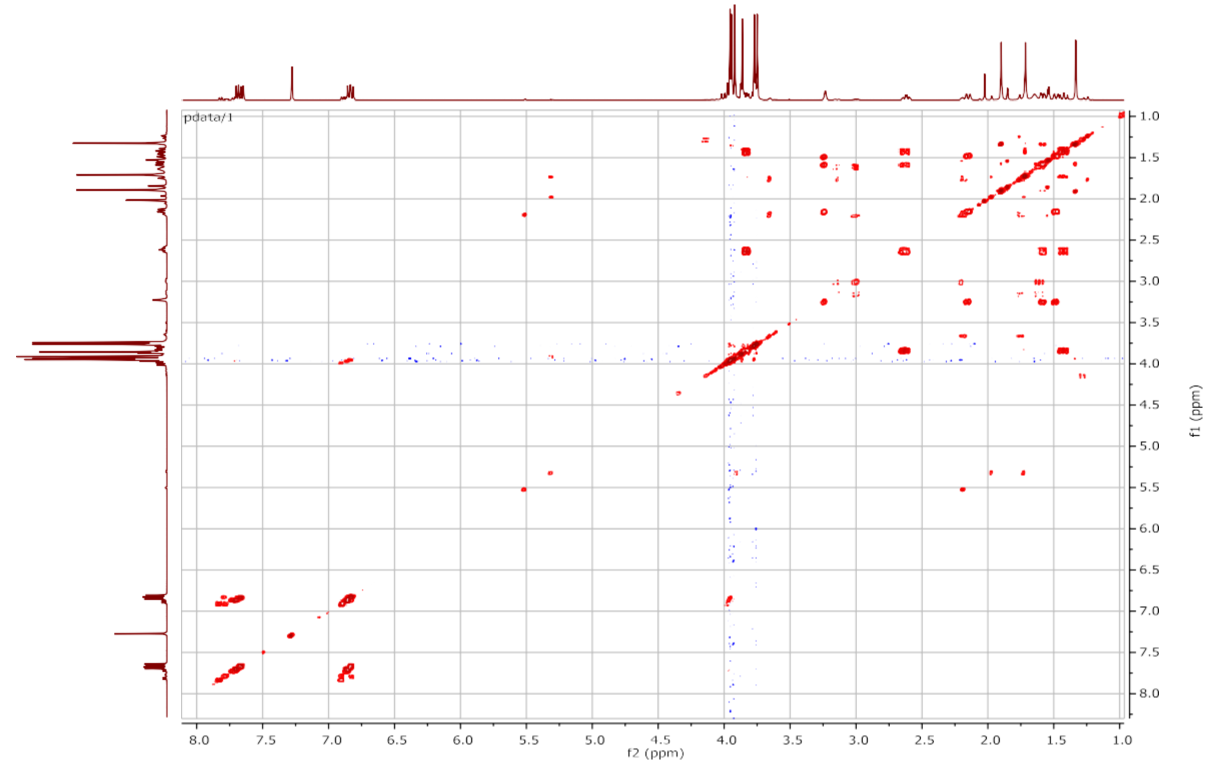

FIGURE S13: ^{1}H-^{1}H COSY spectrum of compound **4**

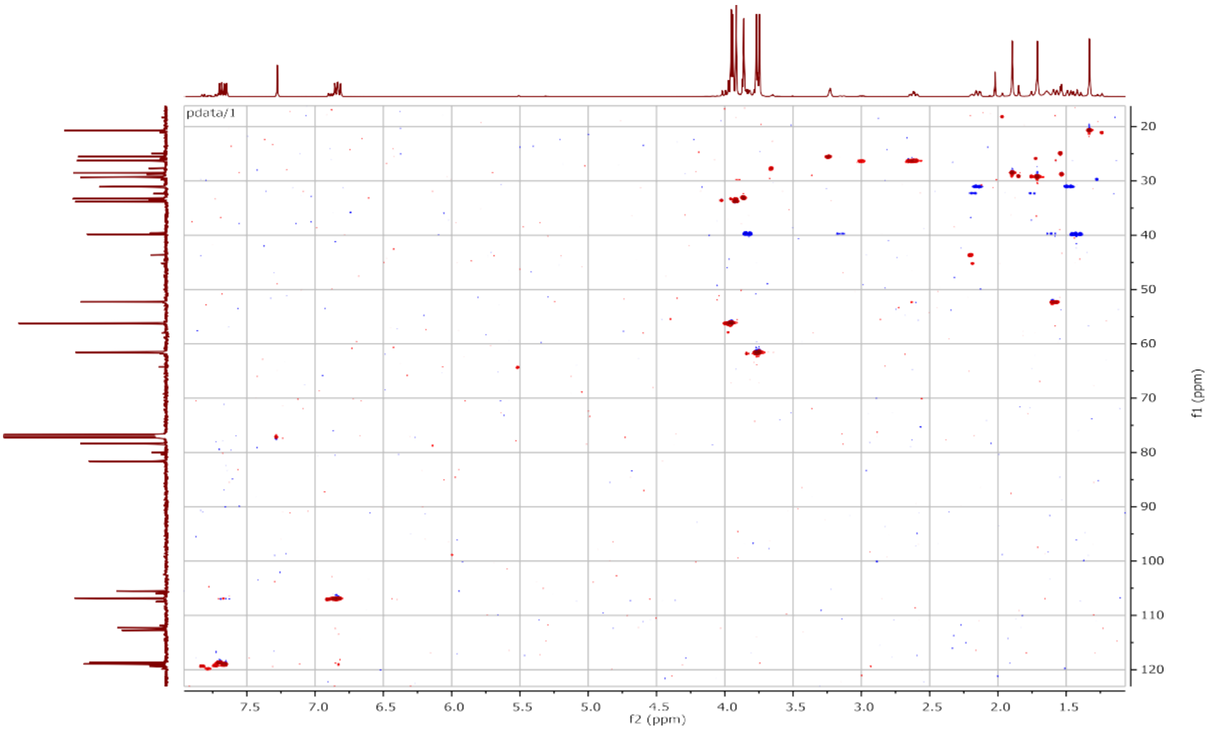

FIGURE S14: HSQC spectrum of compound **4**

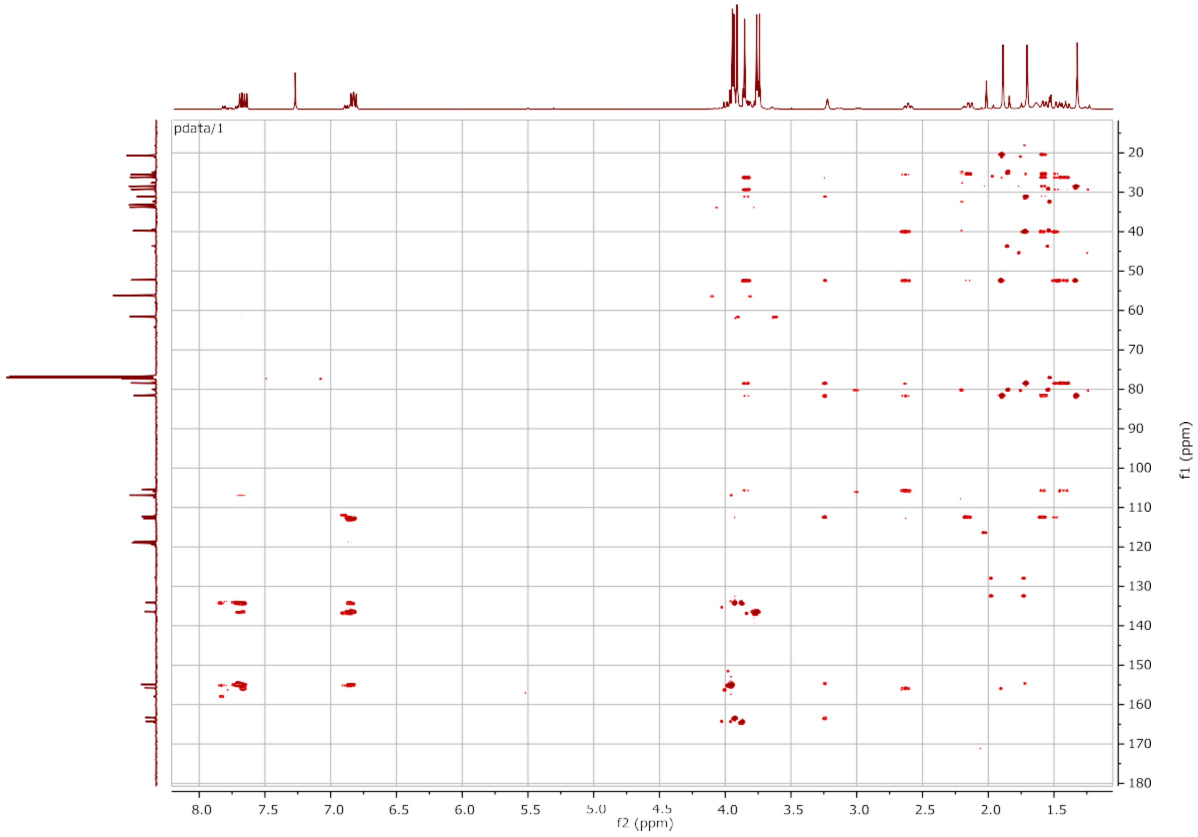

FIGURE S15: HMBC spectrum of compound **4**

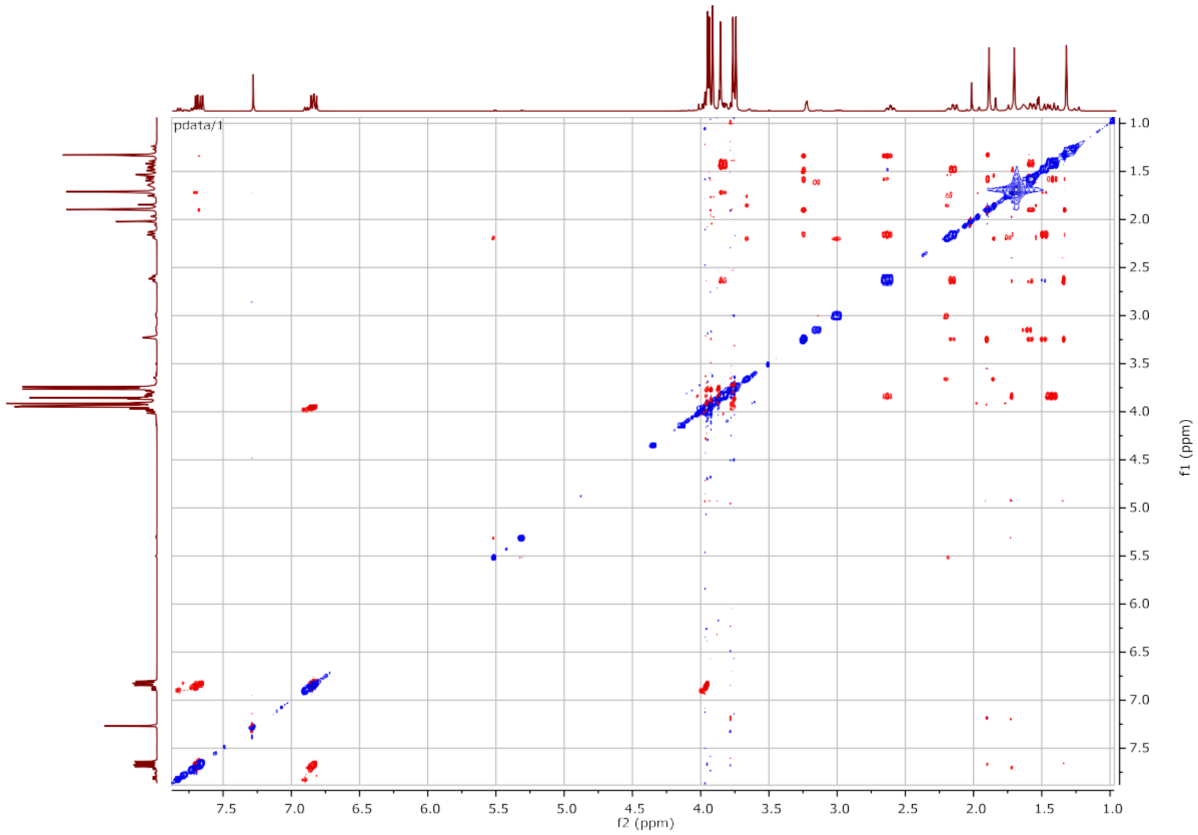

FIGURE S16: NOESY spectrum of compound **4**

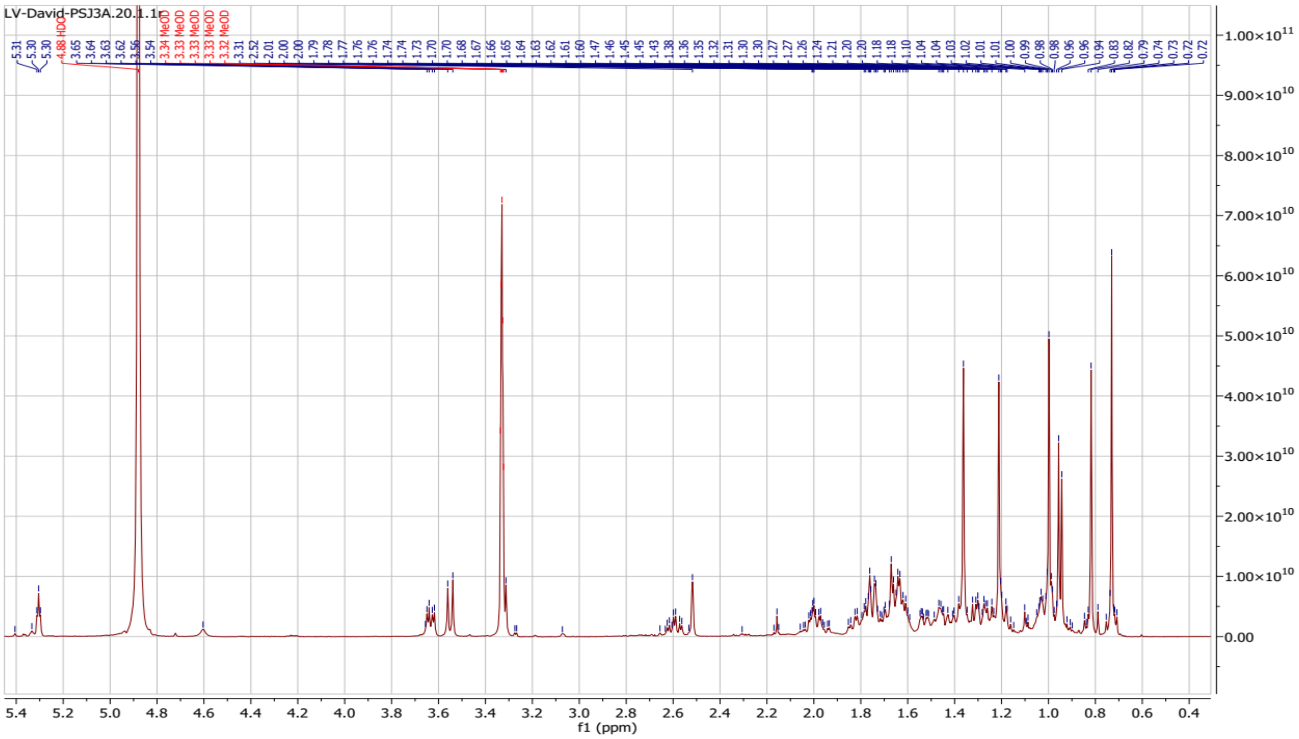

FIGURE S17: ^{1}H NMR spectrum of compound **5**

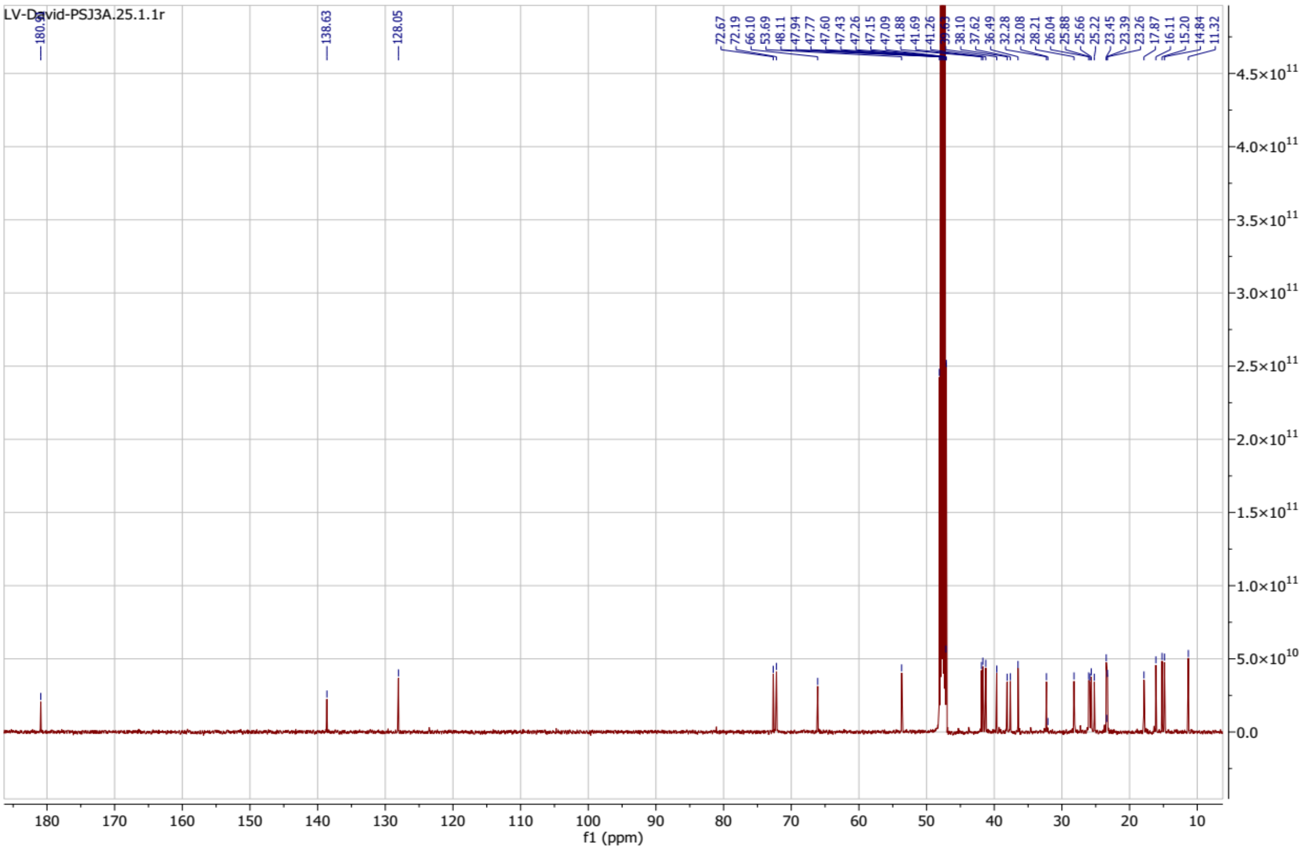

FIGURE S18: ^{13}C NMR spectrum of compound **5**

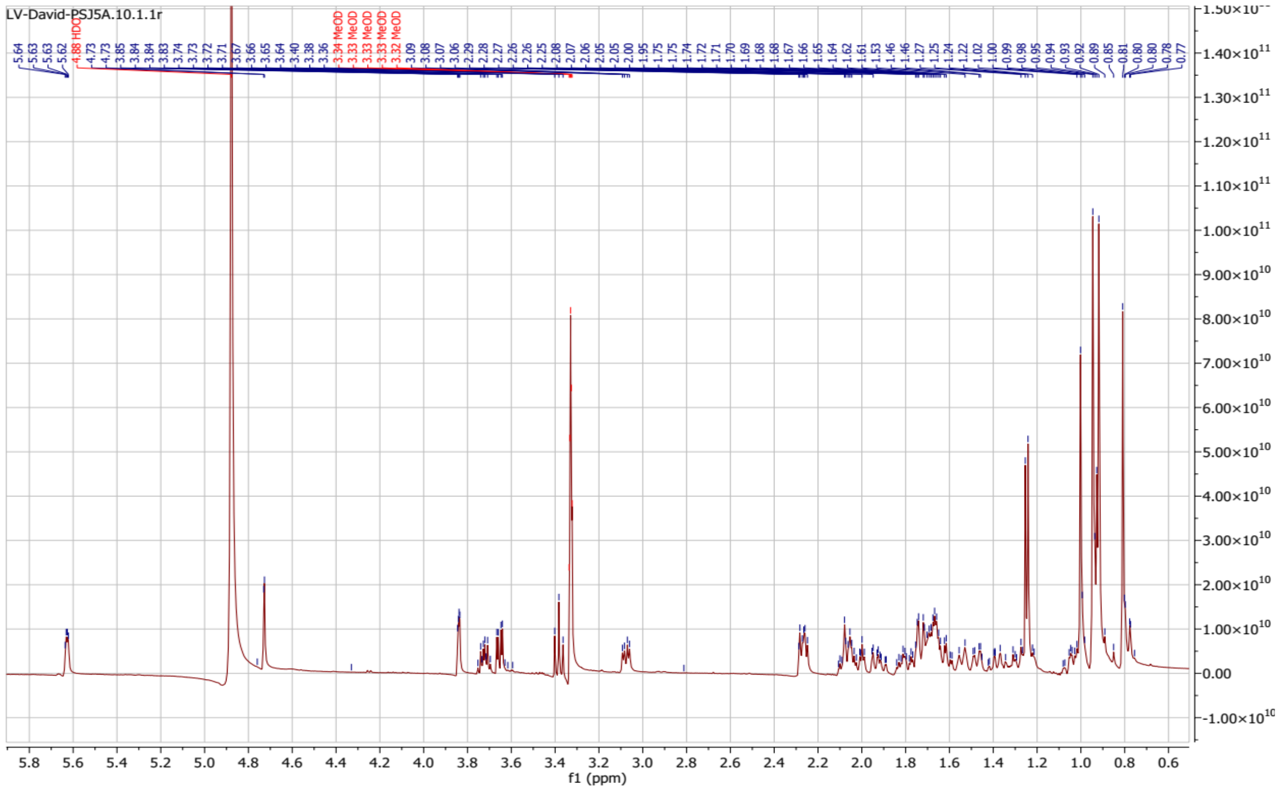

FIGURE S19: ^{1}H NMR spectrum of compound **6**

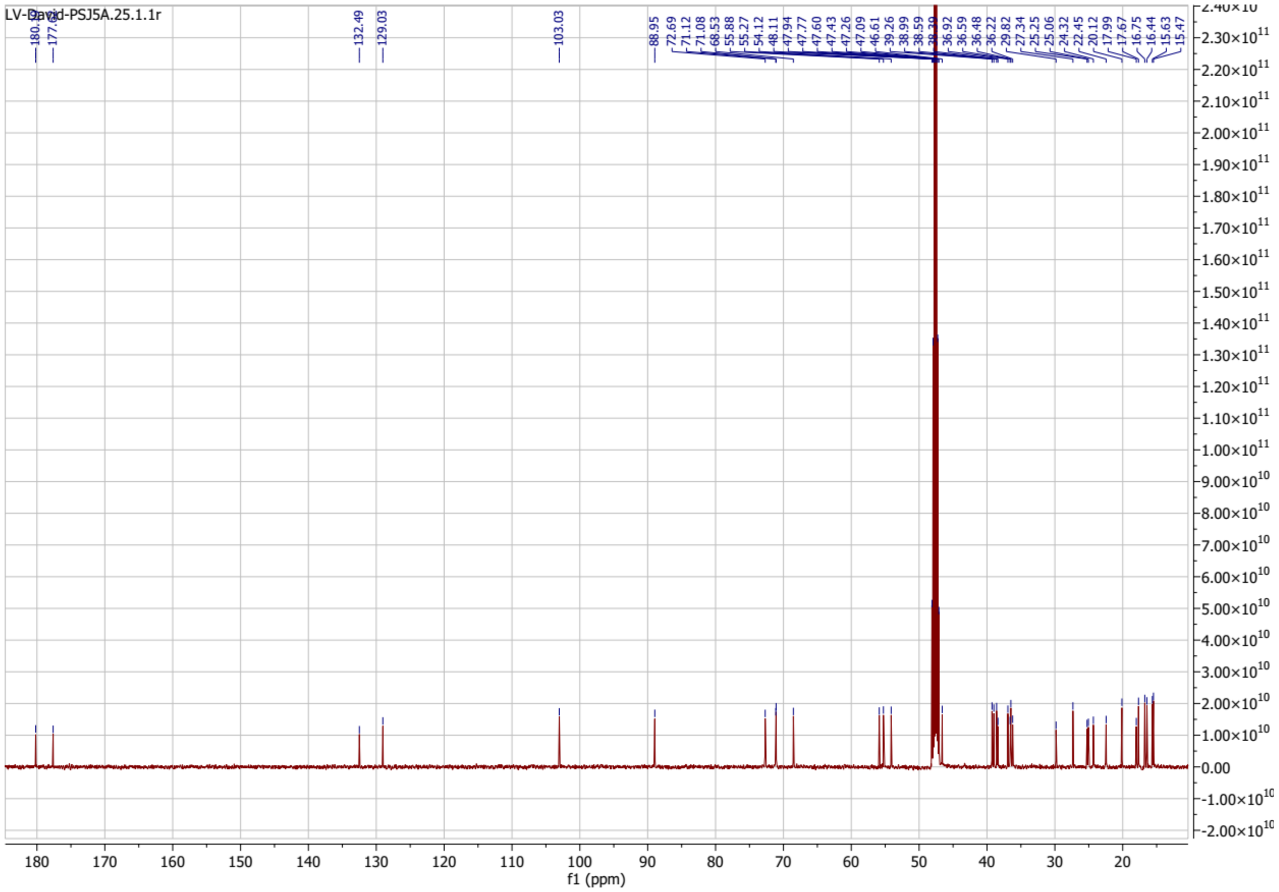

FIGURE S20: ^{13}C NMR spectrum of compound **6**

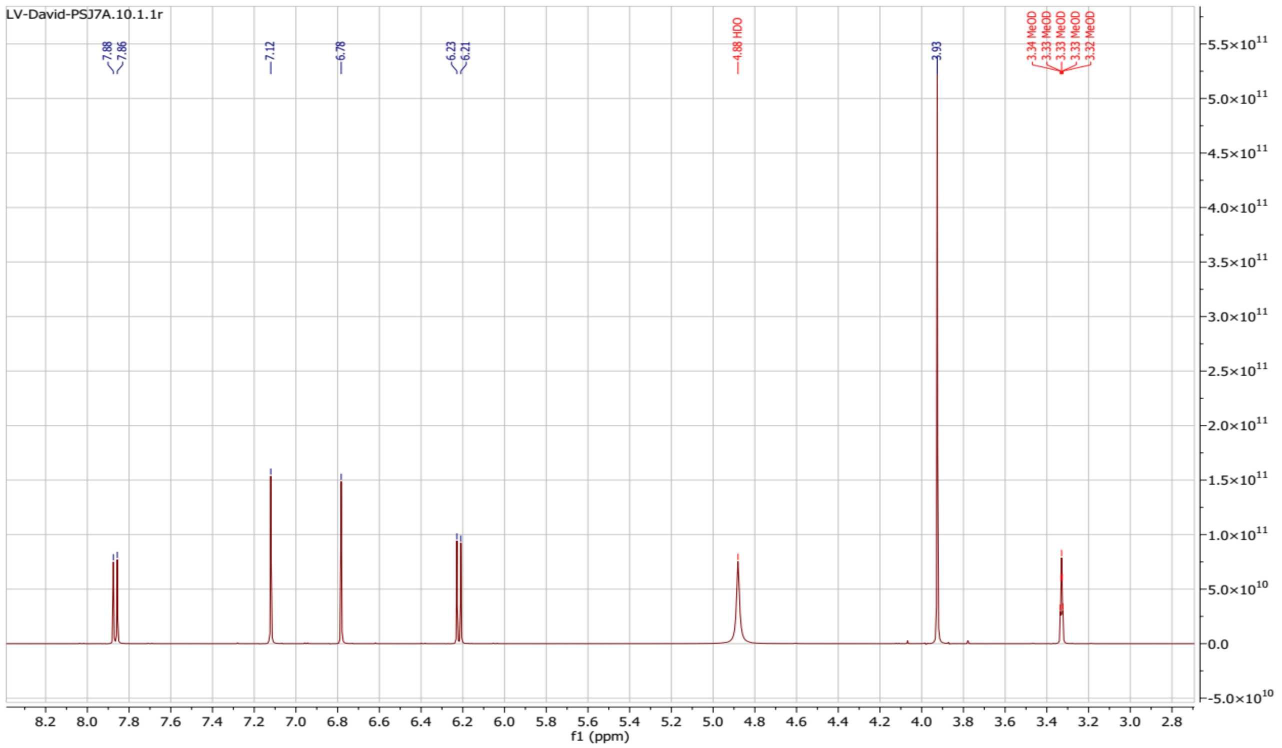

FIGURE S21: ^{1}H NMR spectrum of compound **9**

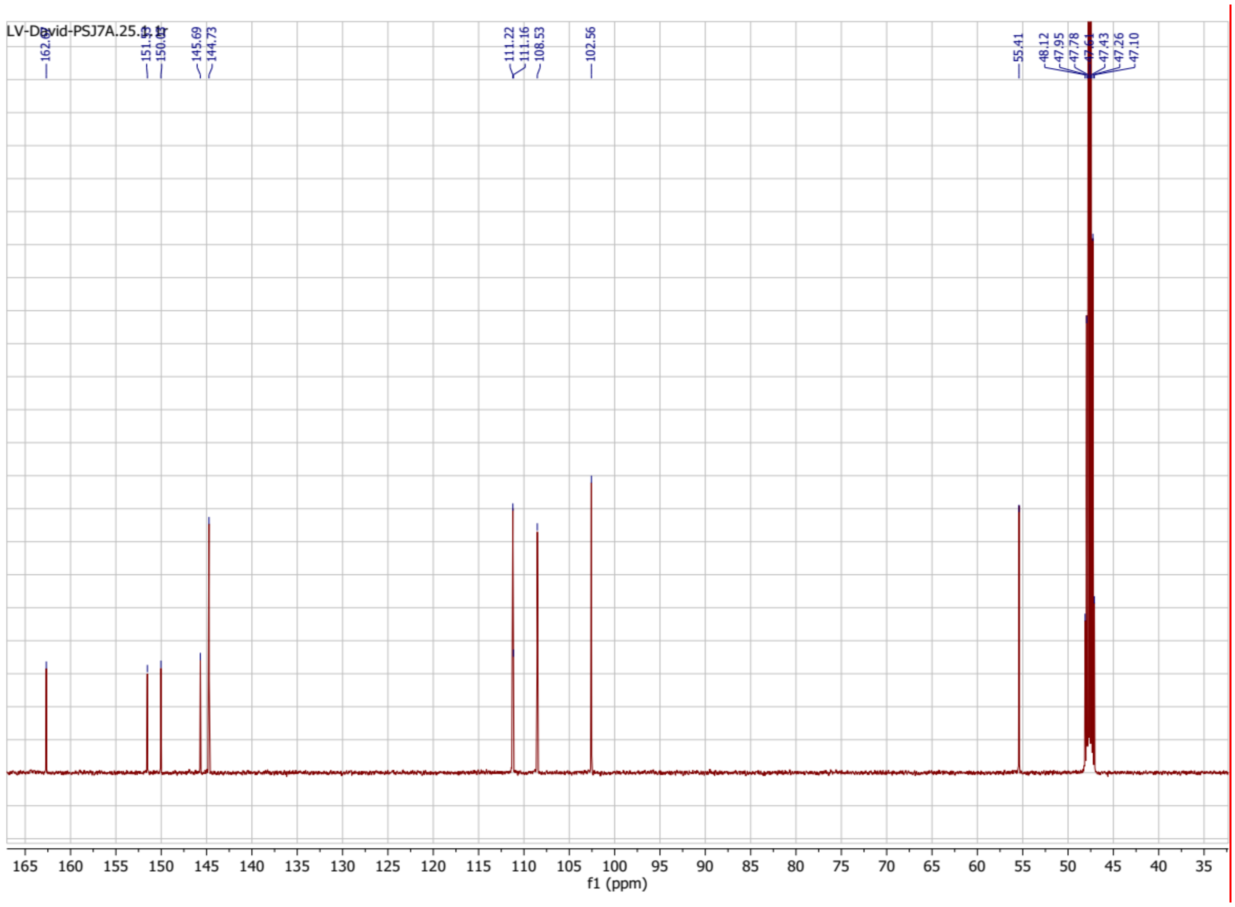

FIGURE S22: ^{13}C NMR spectrum of compound **9**

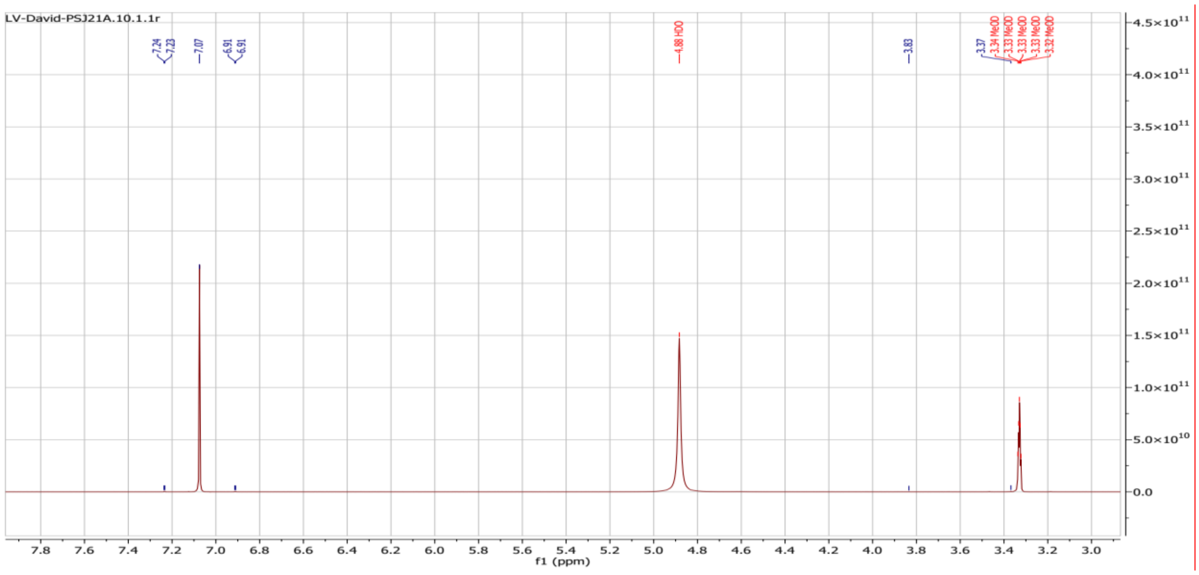

FIGURE S23: ^{1}H NMR spectrum of compound **10**

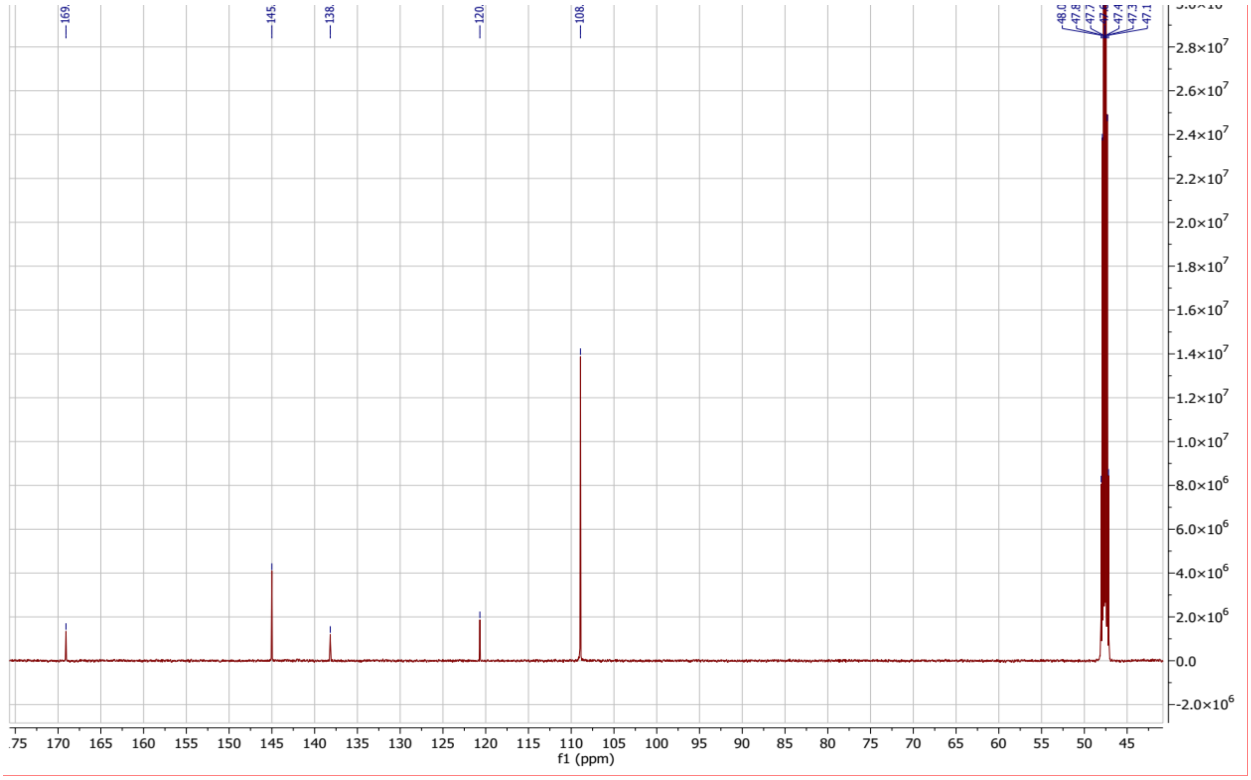

FIGURE S24: ^{13}C NMR spectrum of compound **10**

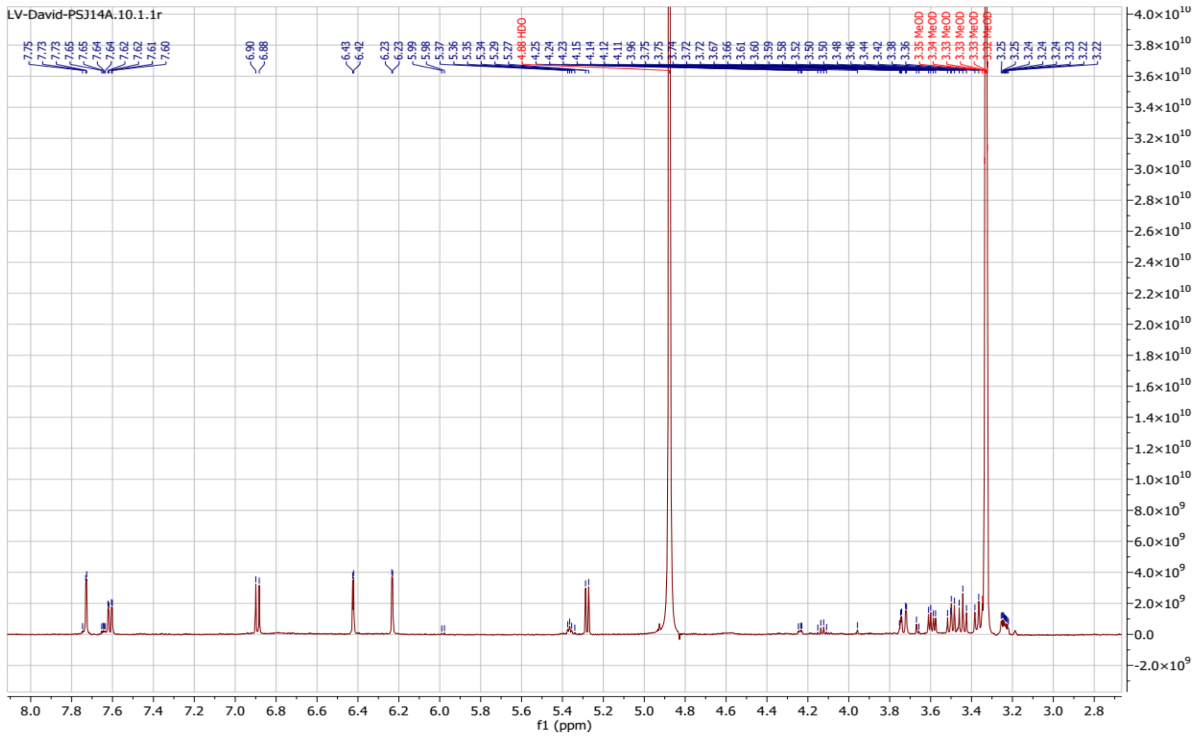

FIGURE S25: ^{1}H NMR spectrum of compound **11**

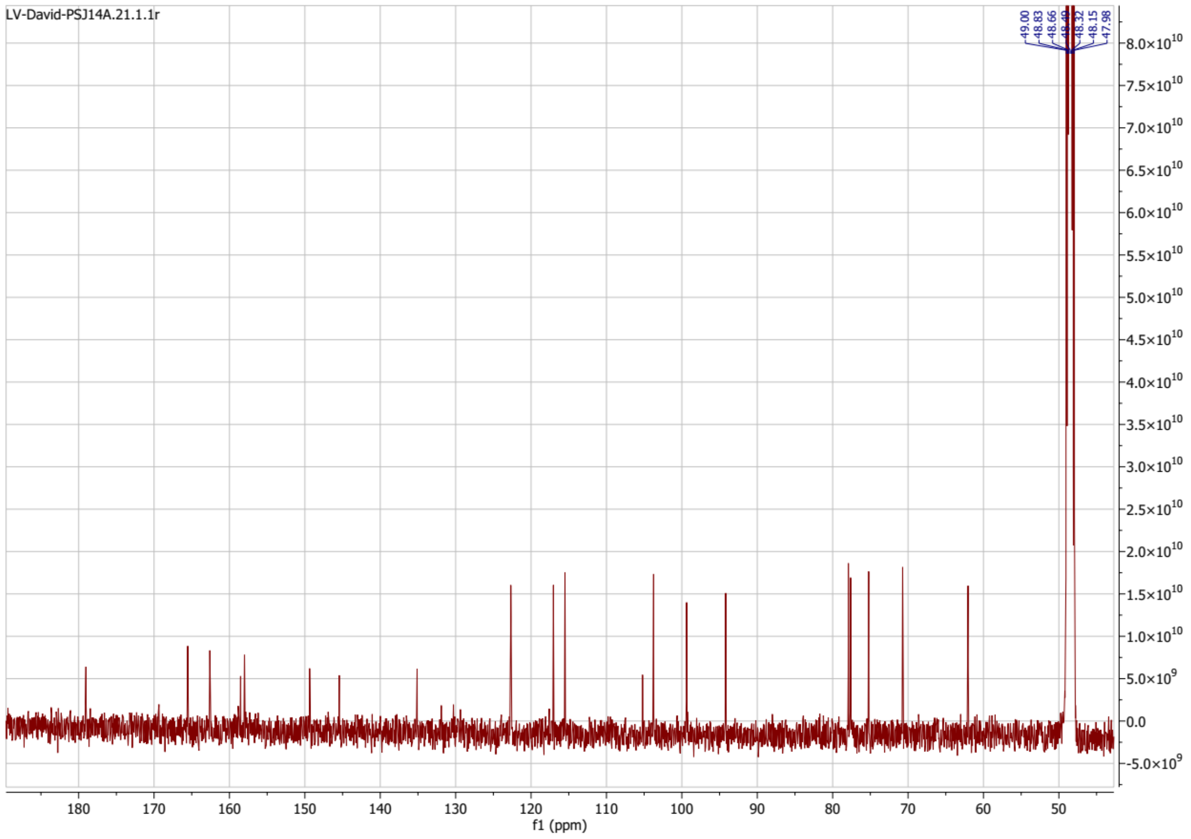

FIGURE S26: ^{13}C NMR spectrum of compound **11**

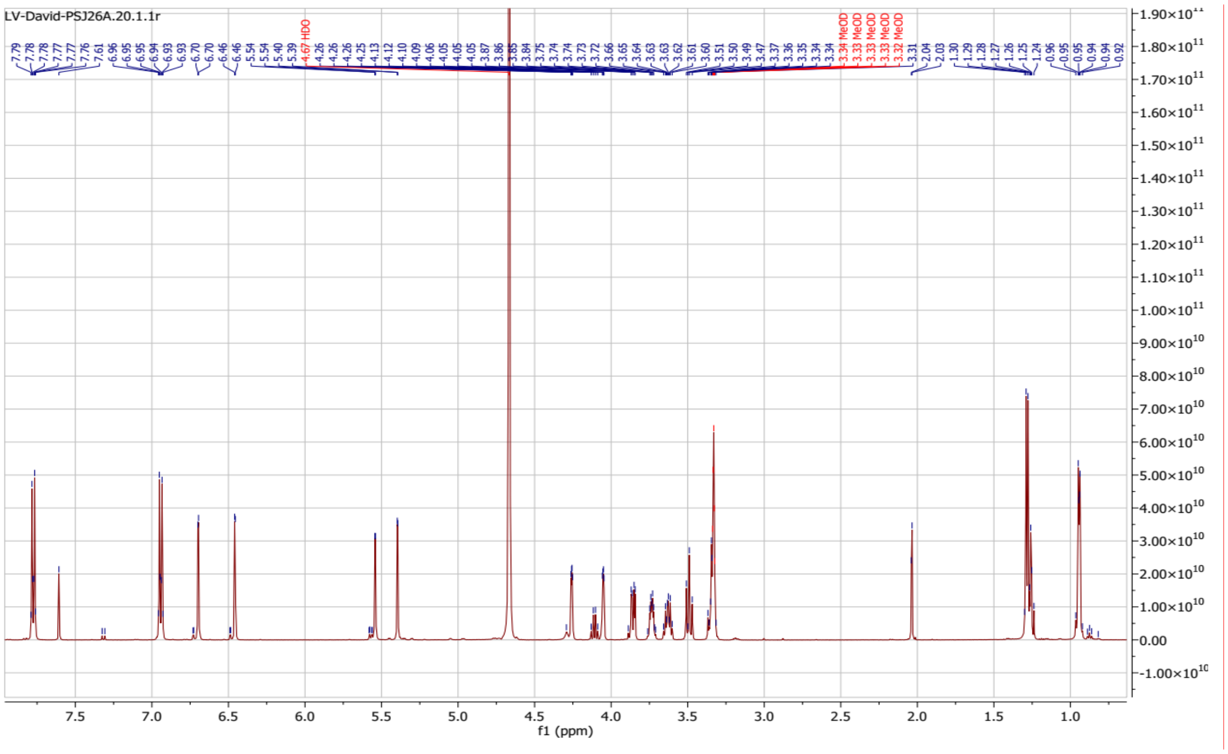

FIGURE S27: ^{1}H NMR spectrum of compound **12**

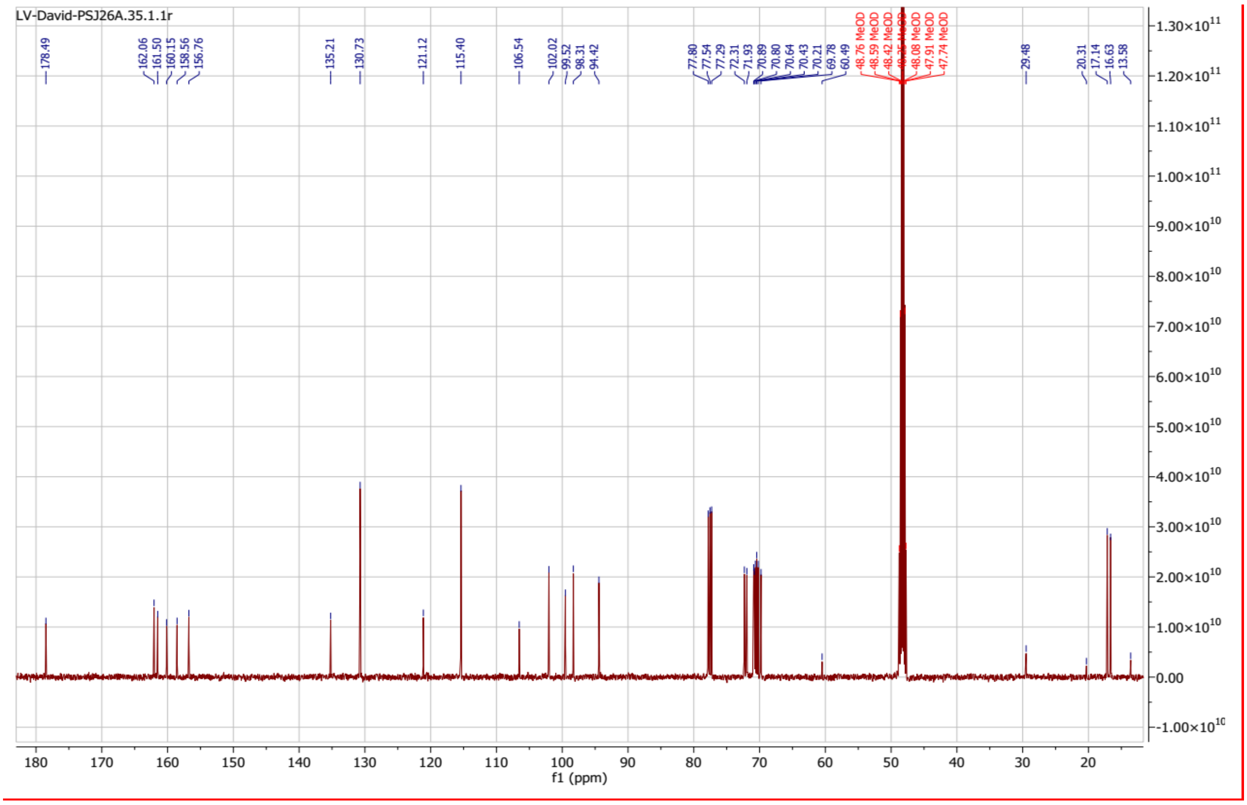

FIGURE S28: ^{13}C NMR spectrum of compound **12**

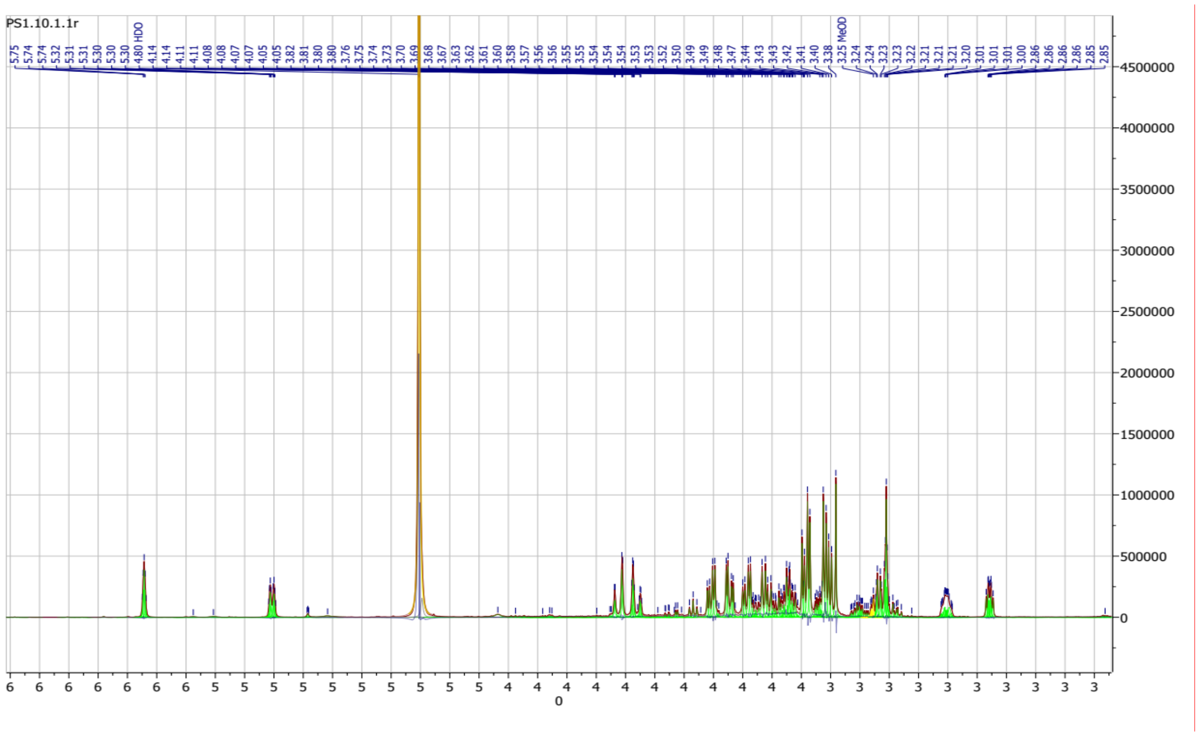

FIGURE S29: ^{1}H NMR spectrum of compound **13**

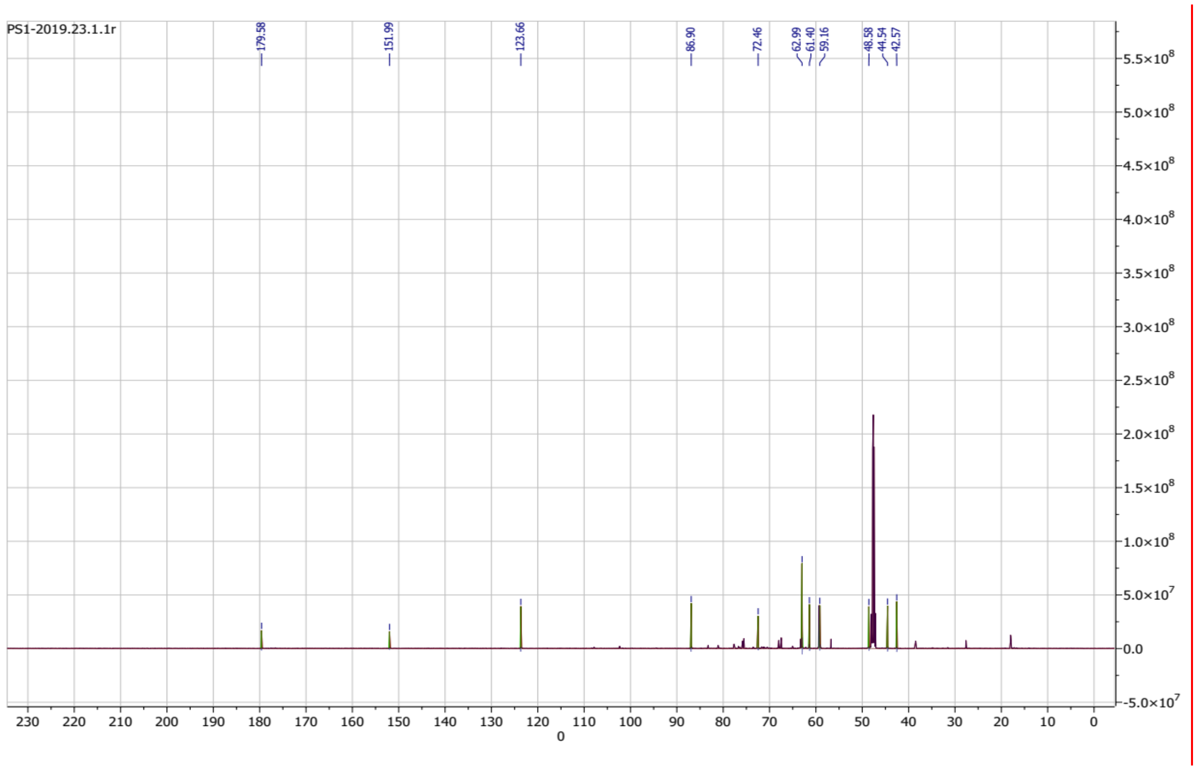

FIGURE S30: ^{13}C NMR spectrum of compound 1**3**

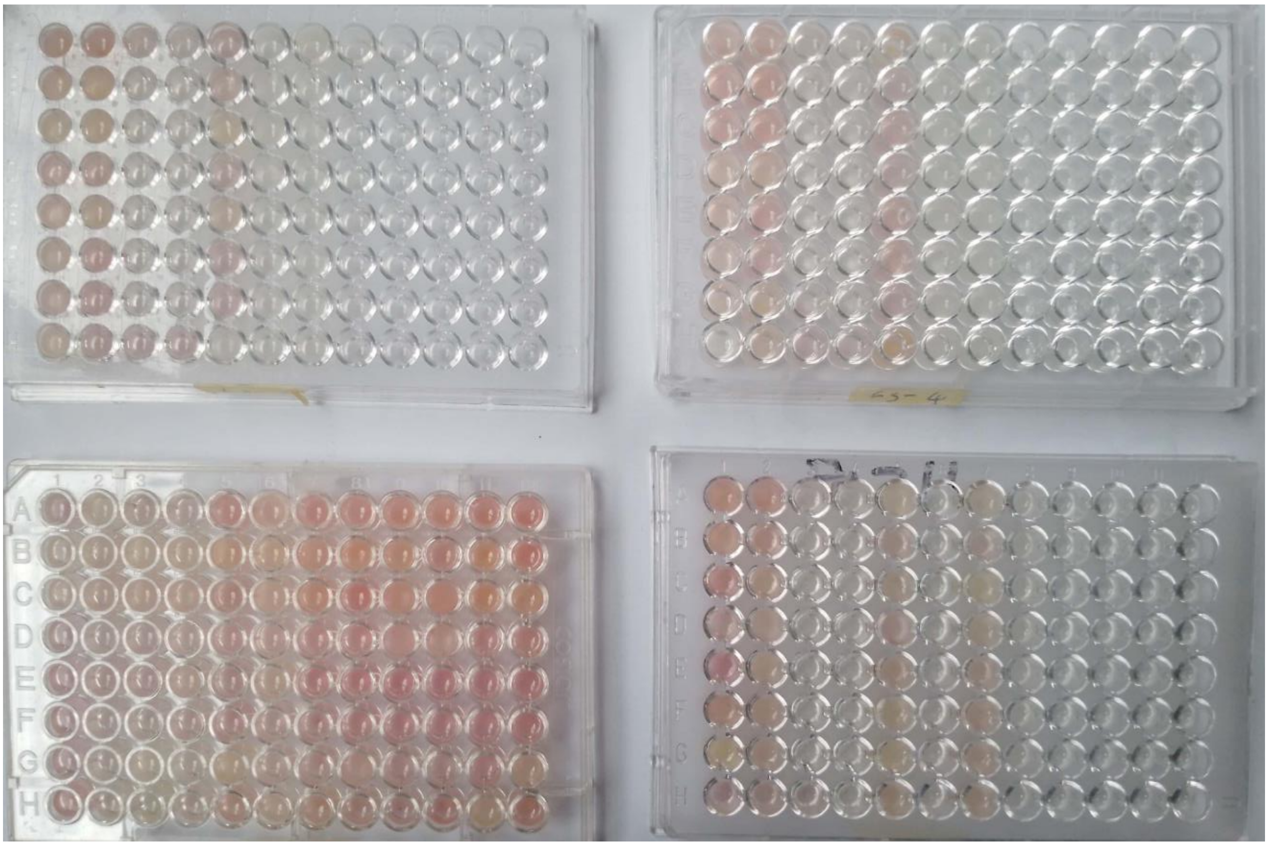

FIGURE S31: Microtiter plate images after INT colorimetric assay.

The viability of bacterial cells after treatment with extracts/compounds was determined by the INT assay. Viable bacteria produced a pink coloration from the initial yellow one. The smallest sample concentration values (i.e. MIC) that altered this color change was considered to have offered maximum inhibition of bacterial growth.
